# The Evolution of Gene Sequencing Technologies: Unveiling Genetic Architecture of Nonsyndromic Orofacial Clefts

**DOI:** 10.1155/genr/3754674

**Published:** 2026-04-22

**Authors:** Haolang Zhao, Sidi Zhang, Haoyun Zhu, Zhonglin Jia

**Affiliations:** ^1^ Department of Cleft Lip and Palate Surgery, State Key Laboratory of Oral Diseases, National Center for Stomatology, National Clinical Research Center for Oral Diseases, West China Hospital of Stomatology, Sichuan University, Chengdu, 610041, China, scu.edu.cn

**Keywords:** next-generation sequencing, nonsyndromic orofacial clefts, sequencing technologies, susceptibility genes

## Abstract

Nonsyndromic orofacial clefts (NSOC) are common congenital craniofacial developmental defects. Current evidence suggests that genetic factors, environmental exposures, and their interactions jointly contribute to the development of the disease. Owing to the high heritability of NSOC, identifying susceptibility genes and loci is a central focus of etiological research. This review summarizes key findings in the identification of NSOC susceptibility genes and loci across successive stages of sequencing technology development. With the evolution of sequencing approaches, from Sanger sequencing to next‐generation sequencing (NGS) and third‐generation sequencing (TGS), and more recently to emerging technologies including epigenomics, single‐cell sequencing, spatial omics, and multiomics integration, the field of NSOC genetics has undergone a transformative shift from low‐throughput to high‐throughput analyses. These advancements have enabled progress from the identification of common, classical susceptibility genes to the discovery of *de novo* mutations, rare variants, complex genomic structural variations, and the elucidation of cell differentiation trajectories. These advances have substantially enhanced our multidimensional understanding of the genetic heterogeneity underlying NSOC and reflect a broader transition in research focus from susceptibility mapping to mechanistic elucidation. Future studies should continuously promote methodological innovations in sequencing technologies, optimize study design, and explore integrative multiomics approaches to refine ethnicity‐ and subtype‐specific genetic databases. Accelerating the translation of basic research findings into clinical applications will provide a solid foundation for early disease screening, genetic counseling, and precision prevention.

## 1. Introduction

Nonsyndromic orofacial clefts (NSOC) are prevalent congenital malformations of the maxillofacial region, with a global prevalence of approximately 1 in 800. This prevalence varies considerably across different ethnicities, geographical regions, and genders [1]. Similar to most complex genetic disorders (e.g., congenital heart disease and autism spectrum disorder) [2], the etiology underlying NSOC is highly intricate, involving genetic susceptibility, environmental exposures, and their interactions [3]. Consequently, traditional candidate gene approaches fall short of fully elucidating the pathogenic mechanisms of this condition.

Furthermore, NSOC exhibit distinct clinical phenotypic heterogeneity [4]. Based on the affected anatomical sites, NSOC is generally categorized into three major subtypes: nonsyndromic cleft lip only (NSCLO), nonsyndromic cleft palate only (NSCPO), and nonsyndromic cleft lip and palate (NSCLP). Although a multidisciplinary sequential treatment paradigm for NSOC has been established, the adverse outcomes induced by the malformation, including dysfunctions such as impaired feeding, speech, and hearing, along with the subsequent psychological distress and difficulties in social adaptation, cannot be completely corrected or cured [1].

**FIGURE 1:**
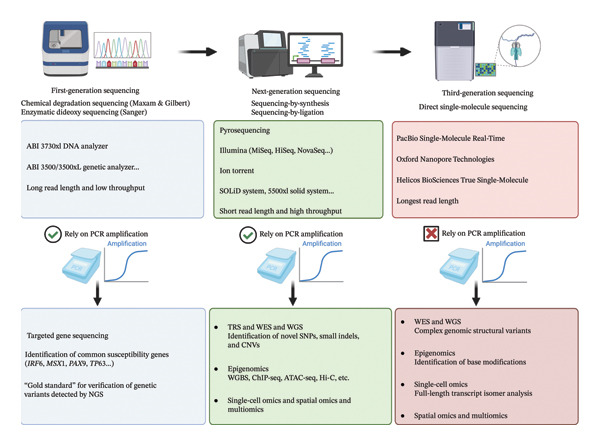
The evolution and comparison of different sequencing technologies in NSOC genetic research over the past few years, along with the primary applications of each sequencing method.

Therefore, an in‐depth investigation into the pathogenesis of NSOC is of great significance for facilitating early screening, genetic counseling, and precise prevention. Over the past decades, gene sequencing technologies have undergone rapid development. They started with first‐generation sequencing, which is dominated by Sanger sequencing, and have advanced to include various high‐throughput sequencing approaches, such as targeted region sequencing (TRS), whole‐exome sequencing (WES), and whole‐genome sequencing (WGS). These technological advances have provided robust technical support for deciphering the pathogenic mechanisms of NSOC and other complex genetic disorders [5, 6]. Figure 1 illustrates the evolution of sequencing technologies applied in NSOC research in recent years, as well as the core characteristics or major research achievements of each sequencing method.

This review article recapitulates the developmental course of gene sequencing technology at different stages. We specifically focus on how technological innovations in gene sequencing have enabled genetic research on NSOC to evolve from the detection of single genes and variants to the comprehensive exploration of novel susceptibility variants on a genome‐wide scale.

## 2. Sanger Sequencing Drives the Identification of Common Susceptibility Genes in NSOC

### 2.1. Sanger Sequencing

Sanger sequencing, developed in 1977, is based on the dideoxy chain‐termination method proposed by Frederick Sanger. This method separates and detects DNA fragments of different lengths generated from the same DNA template using capillary electrophoresis and is, therefore, considered as the first‐generation sequencing technology. Sanger sequencing not only laid the foundation for modern DNA sequencing technologies [7] but has also been considered the “gold standard” for gene sequencing because of its high accuracy [8].

### 2.2. Application Approaches

#### 2.2.1. Candidate Gene–Targeted Sequencing

During the era of Sanger sequencing, studies investigating the pathogenesis of NSOC predominantly adopted a candidate gene sequencing. Based on existing evidence from the literature, including that derived from animal models and analyses of known signaling pathways involved in craniofacial development, researchers selected candidate genes potentially associated with NSOC. Targeted gene sequencing was performed on the coding regions or regulatory elements of these genes to screen for susceptibility variants. Using this strategy, several novel susceptibility genes and risk‐associated variants for NSOC were successfully identified, such as point mutations in *FOXE1*, *GLI2*, *JAG2*, *SPRY2*, and *RYK* [9, 10]. However, the limited sequencing coverage of this approach restricted the discovery of additional susceptibility genes and loci [11].

#### 2.2.2. Mutation Screening and Cosegregation Analysis in Pedigrees

Family‐based cosegregation analysis is the key genetic sequencing for validating the pathogenicity of candidate variants. In pedigrees with a family history of disease, candidate genes are sequenced in multiple family members across generations. By combining pedigree structure with phenotypes, this method assesses whether a detected variant is present exclusively in affected individuals and absent in unaffected members, thereby determining whether the inheritance pattern of the candidate variant is consistent with the transmission of the disease within the family [12]. When a variant strictly cosegregates with the disease phenotype, where all affected individuals carry the variant and all unaffected individuals do not, strong genetic evidence is provided to support its pathogenic role. Following the Mendelian inheritance principles, this strategy has successfully identified several important susceptibility genes for cleft lip and palate, including interferon regulatory factor 6 (*IRF6*), msh homeobox 1 (*MSX1*), and *PVRL1*, making significant contributions to the genetic studies of orofacial clefts [13, 14].

#### 2.2.3. Case–Control Population Screening

Case–control screening is an important strategy for applying candidate gene approaches at the population level. In this approach, Sanger sequencing is performed on the coding regions or specific functional sites of candidate genes among NSOC cases and healthy controls. By comparing allele frequency differences between the two groups, the strength of association between genetic variants and NSOC risk can be evaluated [9]. One major advantage of case–control screening is its ability to assess the population distribution of specific variants and quantify their contribution to the disease risks. Compared with family‐based studies, this method does not rely on large pedigrees, which is easier to implement, and is particularly suitable for investigating the effects of common variants (allele frequency > 1%). As a result, it has yielded important findings in NSOC genetic research [15–17].

However, case–control screening results may be influenced by factors such as the sample size, population stratification, and environmental confounders [1, 18]. Therefore, careful study design, appropriate case–control matching, and rigorous statistical analyses are essential. Moreover, findings should be validated in independent external cohorts to ensure their robustness and reliability [19].

### 2.3. Major Findings From Sanger Sequencing in the Identification of NSOC Susceptibility Genes

#### 2.3.1. *IRF6*

The *IRF6* plays a critical role in regulating epithelial cell differentiation, migration, apoptosis [20], modulating neural crest cell function [21], and craniofacial bone formation through multiple signaling pathways [22]. In 2002, Kondo et al. identified *IRF6* as the primary pathogenic gene causing Van der Woude syndrome (VWS) through direct sequencing analysis [23]. In 2004, a large‐scale study involving 8003 individuals from Asian, European, and South American populations used transmission disequilibrium testing and case–control analyses to firstly demonstrate an association between the V274I polymorphism of the *IRF6* gene (rs2235371, c.820G > A) and NSOC risk [24]. This variant results in a valine‐to‐isoleucine substitution at the amino acid position 274. The association of this locus was subsequently confirmed in an independent NSCL/P family‐based study [25]. However, findings on the rs2235371 were controversial. Some studies suggest that this variant has limited direct functional effects and may influence NSOC susceptibility through specific haplotypes (e.g., rs2235371G/rs642961A and rs2235371C/rs642961G) [26, 27]. Moreover, the effect of this locus appears to be strongly influenced by population genetic background [28], highlighting the need for further validation across diverse populations. In addition, Gowans et al. identified four novel variants, including p.Glu69Lys and p.Asn185Thr, associated with NSOC in African populations [29]. Pegelow et al. further demonstrated the potential pathogenic effects of variants such as rs2236907 and rs2013162 in NSCL/P among Vietnamese Kinh populations [30].

#### 2.3.2. *MSX1*

The *MSX1* is a member of the muscle segment homeobox gene family. In *MSX1* knockout (*MSX1*−/−) mice, proliferation of palatal mesenchymal cells is markedly reduced, resulting in severe cleft palate and dental developmental defects [31]. Notably, the overexpression of *BMP4* in the palatal mesenchyme can rescue the cleft palate phenotype [31]. As in the 1990s, studies had suggested a potential association between *MSX1* and the risk of NSOC [32–34]. In 2003, Jezewski et al. performed Sanger sequencing in 917 NSOC patients and 1027 controls and demonstrated that *MSX1* mutations were pathogenic factors contributing to NSOC [35]. A family‐based case–control study in a Nigerian population further identified a missense mutation (A34G) at rs36059701 associated with cleft lip and palate [36]. This association may be attributable to the disruption of the N‐terminal polyproline tract of the *MSX1* protein, thereby impairing its transcriptional repressor function. In the Malay populations, carriers of the *MSX1* rs8670 (C > T) polymorphism were found to have a reduced risk of NSCL/P, with the T allele potentially exerting a protective effect by influencing mRNA stability [37]. In addition, multiple studies across diverse populations have consistently demonstrated significant associations between *MSX1* mutations and NSOC susceptibility [32, 38]. More recently, *MSX1* polymorphisms have been shown to be significantly associated with tooth agenesis among patients with NSCL/P in South Indian populations [39].

#### 2.3.3. Paired box 9 (*PAX9*)

*PAX9*, a member of the PAX gene family, functions as a key transcription factor governing craniofacial and tooth development. In *PAX9* knockout (Pax9^−/−^) mice, the expression of the Wnt signaling inhibitors is significantly upregulated in the palatal mesenchyme, whereas Wnt ligands are downregulated. This leads to insufficient elongation, delayed elevation, and fusion failure of the palatal shelves [40]. Chinese researchers screened for genetic variants in the *PAX9* gene among 180 Han Chinese patients with NSCPO from western China, identifying two significantly associated polymorphic loci, rs12885612 and rs12881248. Notably, these two single‐nucleotide polymorphisms (SNPs) exhibited a significant correlation with NSCLO in subsequent association analyses, indicating that they may serve as susceptibility loci for NSCLO [41]. Another study focusing on the Chinese population revealed a significant association between rs17176643 in *PAX9* and NSCL/P. Moreover, the combination of this locus with rs2073485, rs2235371, or rs2236909 in *IRF6* was found to further elevate the risk of NSCL/P [42].

#### 2.3.4. Tumor Protein 63 (*TP63*)

The *TP63* gene is a member of the p53 protein family, encoding multiple isoforms of the p63 transcription factor. As a key hub of transcriptional and signaling networks in epithelial cells, *TP63* exerts extensive regulatory effects on cellular development, differentiation, and lineage specification [43]. A case–control study conducted on the Han Chinese population focusing on NSCLO demonstrated a significant association between the rs1345186 of *TP63* and the occurrence of right‐sided cleft lip [44]. Furthermore, researchers detected a correlation between the *TP63* c.1324C > T variant and NSCL/P in a Chinese pedigree [45]. This mutation introduces a premature termination codon (p.Q442^∗^), which in turn impairs the oligomerization capacity of the p63 protein.

In addition to the aforementioned representative genes and loci, Sanger sequencing has also confirmed associations between genetic variants in genes including *MTHFR* [28, 46], *VAX1* [17], *PBX1* [47], *PAX7* [48], *ARHGAP29* [49], *PARD3* [50], *ACSS2* [15], *GREM1* [51], *BMP4* [52], *PVRL1* [53], *CDH1* [54], and susceptibility to NSOC.

#### 2.3.5. Limitations

As the first‐generation DNA sequencing technology, Sanger sequencing has provided crucial technical support for molecular genetic studies of NSOC owing to its high accuracy and long‐read length characteristics. However, as genomic research expands toward large‐scale population genetic analysis and genome‐wide investigations, the limitations of this technology have become increasingly prominent: (i) its cumbersome and time‐consuming operational procedures lead to high costs and entail the risk of experimental errors [55] and (ii) its limited throughput fails to meet the demands of large‐scale sample screening or complex genomic research [56]. These drawbacks are particularly pronounced in NSOC, a condition marked by high genetic heterogeneity [57], and have directly driven the widespread adoption of high‐throughput next‐generation sequencing (NGS) technology [58]. In the NGS era, Sanger sequencing has evolved into an auxiliary validation tool, which is employed for the independent verification of key genetic variants, thereby providing complementary evidence for genetic studies of NSOC [59].

## 3. Emergence of NGS and Large‐Scale Genomic Discovery

In contrast to the Sanger sequencing, NGS technologies, which emerged in the early 21st century, are based on the principle of sequencing‐by‐synthesis (represented by Illumina and Ion Torrent) or sequencing‐by‐ligation (represented by ABI SOLiD) [60, 61]. These technologies enable massively parallel sequencing of millions of DNA fragments simultaneously. This technological innovation has substantially increased sequencing throughput and speed while significantly reducing sequencing costs [57].

The core advantages of NGS include high coverage, high sensitivity and broad detection capacity [62]. NGS platforms can be applied across multiple scales, ranging from single‐gene analysis and targeted gene panels to WES and WGS, thereby extending NSOC research from early candidate gene studies to comprehensive, genome‐wide screening. Importantly, NGS enables efficient detection of various genetic variations, including single nucleotide variants (SNVs), small insertions/deletions (indels), and structural variants (SVs) (especially copy number variants [CNVs]) [5, 63]. This approach enables thousands of genes to be examined within a single experiment, providing novel opportunities to elucidate the molecular mechanisms underlying complex genetic disorders such as NSOC.

With the advancement of NGS, three major sequencing strategies have been progressively established: TRS, WES, and WGS. These approaches reflect a methodological transition in NSOC genetic research, which rooted from candidate gene validation to the discovery of novel susceptibility genes, and ultimately to the investigation of complex regulatory mechanisms across the genome [64].

### 3.1. TRS

#### 3.1.1. Technical Characteristics

High‐throughput TRS was first reported by Albert et al. in 2007 [65]. This approach selectively captures specific functional regions of the genome using probe hybridization or PCR‐based enrichment, combined with high‐throughput sequencing on NGS platforms. By increasing sequencing depth within targeted regions, TRS markedly improves sensitivity and accuracy while substantially reducing costs. Following optimization by the Hodges and Gnirke groups, TRS has become one of the earliest NGS‐based strategies to be translated into practical application [66, 67].

Compared with traditional sequencing methods, the major advantage of TRS is its ability to analyze multiple samples and multiple genes simultaneously. High‐depth coverage enables efficient detection of the majority of variants within target regions, including low‐frequency and rare variants [68], making this approach particularly suitable for diseases with high genetic heterogeneity. In addition, TRS is characterized by cost‐effectiveness, low data‐processing burden, and high flexibility in panel design, which further supported its widespread use in genetic studies [69].

#### 3.1.2. Applications of TRS in NSOC

According to research objectives, TRS can be broadly categorized into three types. Chromosomal region‐targeted sequencing focuses on defined chromosomal segments to investigate the functional roles of regulatory elements [70]. Using targeted sequencing of the 2p21 region, Li et al. identified *ZFP36L2* as a novel susceptibility gene for NSCLO, as *ZFP36L2* potentially influenced disease risk through regulation of cell proliferation and the cell cycle [71]. Pan et al. performed targeted sequencing of the 1p22 region and identified rs77179923 (*ABCA4*) and rs12071152 (located in the intergenic noncoding region) as variants specifically associated with NSCL/P and left‐sided cleft lip, respectively, in the Chinese Han population. Their study further demonstrated that a rare missense variant in *ARHGAP29* (c.1652G > C, p.Arg551Thr) may affect NSCL/P‐related biological processes by downregulating gene expression [72].

In addition, Bartusel et al. conducted targeted sequencing of the enhancer region e2p24.2 and, in combination with the genome‐wide association studies (GWASs) data, showed that this enhancer regulates the expression of *MYCN* and *DDX1* through long‐range chromatin interactions. Alterations in this regulatory element were shown to influence NSOC risk via tRNA splicing [70].

Single‐gene targeted sequencing enables high‐depth coverage sequencing of the candidate susceptibility genes, allowing the comprehensive detection of genetic variants and validation of their associations with disease. Jia et al. performed haplotype‐region sequencing of the *FOXE1* gene in the NSCL/P patients among western Chinese Han population and identified 76 SNVs and 50 insertions/deletions (indels). Association analysis revealed a highly significant association between rs13292899 and NSCL/P risk (*p* = 1.85 × 10^−27^) [73]. Lin et al. sequenced the *KLF4* gene and identified rs6477574, rs112488950, and rs111357138 as significantly associated with elevated NSCL/P risk in the same population [74]. Similarly, Yang et al. sequenced *PAX7* and demonstrated that three SNPs with potential regulatory functions were significantly associated with NSCL/P, while also identifying a novel missense variant, p.Ala369Val (c.1106C > T) [75]. In addition, using TRS, risk variants in *NTN1* [76], *VAX1* [77], and *FAM3B* [78], as well as risk and protective variants in *EPHX2* [79] have been identified in Chinese populations, further expanding the genetic landscape of NSOC.

Multigene panel sequencing employs customized panels comprising multiple functionally related candidate genes to enable high‐throughput, thorough variant screening, thus substantially improving the detection efficiency of susceptibility variants. Dąbrowska et al. designed a targeted panel including 423 genes associated with cleft lip/palate and craniofacial development and screening in NSCPO patients in Poland. This approach led to the identification of eight novel rare variants, including *COL17A1* (c.2435−1G > A) and *DLG1* (c.1586G > C, p.Glu562Asp) [80], providing new insights into the “missing heritability” of NSOC.

### 3.2. WES

#### 3.2.1. Technical Principles and Advantages: Identification of Rare/Low‐Frequency Variants and Discovery of Novel Genes

WES was first applied to human disease research in 2009, where it focused on sequencing the exonic regions (protein‐coding regions) in individuals with a rare dominantly inherited disorder, Freeman–Sheldon syndrome (FSS) [68]. Although the coding region accounts for only approximately 1% of the human genome, it harbors nearly 85% of pathogenic mutations [81, 82]. WES employs sequence‐specific probes to enrich the exonic regions, followed by massively parallel sequencing on high‐throughput platforms, enabling comprehensive detection of SNVs and insertions/deletions (indels) within the coding regions. Originally developed to address the issue of the high cost of WGS, WES has undergone multiple rounds of technical optimization since its first successful application in identifying causative mutations in Mendelian disorders [83], which have substantially improved capture efficiency and sequencing throughput [84].

WES, TRS, and WGS each possesses distinct strengths and form complementary approaches. Compared with TRS, WES provides substantially broader coverage (up to approximately 20,000 protein‐coding genes), which enables an unbiased, genome‐wide exonic scan, making it particularly suitable for cases in which the causative genes are unknown. TRS provides markedly higher sequencing depth within predefined target regions, often reaching 500–1000× coverage, thus offering greater sensitivity for detecting rare variants in known candidate loci. Compared with WGS, WES achieves deep coverage of protein‐coding regions at a lower cost and generates far lower data volumes, substantially simplifying downstream bioinformatic analyses [63, 85]. However, WES fails to detect noncoding variants and large SVs. WES is especially suitable for the discovery of novel disease‐associated genes in pedigrees and for the identification of rare variants following Mendelian inheritance patterns, serving as a critical bridge between TRS and WGS approaches [86, 87].

#### 3.2.2. Representative NSOC Studies

##### 3.2.2.1. Identification of Novel Susceptibility Genes

By providing deep coverage of coding‐region variants, WES enables the detection of disease‐associated loci that are often missed by first‐generation sequencing approaches. A typical example is a study of a Charcot–Marie–Tooth disease type 2 pedigree, in which WES successfully identified a risk variant in *TRPV4* (c.557G > A), whereas prior Sanger sequencing of the same family failed to detect any variants in this gene [88]. In NSOC research, WES has similarly facilitated the identification of multiple novel susceptibility genes, thus broadening the understanding of disease’s genetic background. Alkharafi et al. identified a *DUOX1* mutation (c.4358A > G) in a Kuwaiti family, while this gene was not reported to be associated with NSCL/P [89]. In another study, authors demonstrated for the first time the association between *MMACHC* and NSOC in six Indonesian patients and successfully generated a mouse model exhibiting facial clefts and failure of palatal fusion through knockout of this gene [90]. Notably, Zheng et al. further expanded the genetic spectrum of NSOC via WES, identifying *NRSN2* as a novel causal gene through family‐based WES screening and TRS validation [91]. Mechanistically, wild‐type *NRSN2* negatively regulates TGF‐β signaling by binding to TGF‐β type I/II receptors and promoting their degradation via the endosome–lysosome pathway. The identified loss‐of‐function *NRSN2* variants lose the ability to exert negative regulation on the TGF‐β signaling, leading to the aberrant overactivation of TGF‐β signaling and subsequent NSOC occurrence [91]. Overall, this study provides a rigorous, high‐quality paradigm for WES‐guided genetic etiology research in NSOC.

##### 3.2.2.2. Detection of Rare Variants: Complementarity With GWASs Findings

GWASs have uncovered numerous common risk variants for complex diseases; however, the proportion of heritability explained by these variants remains substantially lower than expectation [92]. Rare variants with high penetrance within families are considered to account for a significant component of this gap, and WES offers distinct advantages for identifying such variants. Copelli et al. performed WES in 18 Brazilian NSOC families and identified a deleterious rare variant in the *FAM193A* gene in one family [93]. Another study on 144 NSOC patients revealed five heterozygous variants in *PTCH2*, p.L104P, p.A131G, p.R557H, p.I927S, and p.V978D, which were rare across multiple public genetic databases [94]. In a cohort of 30 Chinese NSOC hereditary families, Zhao et al. successfully identified 394 rare craniofacial development‐related variants via WES [95]. Researchers further discovered and functionally validated a novel rare biallelic loss‐of‐function variant in *ECPAS* (T644S) as a potential causal mutation for NSOC, providing key evidence for the role of rare variants in the missing heritability of NSOC [95].

##### 3.2.2.3. Identification of De Novo Mutations (DNMs)

The majority of NSOC cases occur sporadically without apparent family history, suggesting that DNMs play an important role in NSOC etiology. Using WES in 22 NSCL/P trios, Chen et al. identified multiple DNMs in genes including *ADGRL2*, *GSC*, *RGPD4*, and *PRSS3* [96]. Protein–protein interaction analyses further demonstrated that those genes harboring DNMs interact with previously known NSCL/P‐associated genes. In another study involving 214 sporadic NSOC patients, *BOC* was found to be a novel susceptibility gene for NSOC, and three missense variants, including p.R407W, p.G436S, and p.D1018N, were identified. This study introduced an “epistatic digenic antagonistic inheritance model,” in which *BOC* p.R681X and *GLI2* p.A543G exert antagonistic effects within the SHH signaling pathway, attenuating the pathogenic impact of *BOC* and resulting in a milder cleft lip phenotype [97].

##### 3.2.2.4. Elucidation of Disease‐Associated Pathways

Functional enrichment analyses of susceptibility genes identified through WES have revealed biological processes and signaling pathways highly relevant to NSOC. Zuo et al. conducted WES in 123 Chinese NSOC families and identified 101 rare pathogenic variants. Enrichment analyses indicated that these genes were significantly associated with primary cilium function, osteogenesis and developmental processes, cell adhesion, and transcriptional regulation [98]. Iovino et al. performed WES followed by gene set enrichment analysis (GSEA) in an NSCPO cohort, which suggested that ultrarare variants were enriched in pathways related to cytoskeletal protein binding, protein complex binding, cell adhesion molecule binding, extracellular matrix (ECM)–receptor interactions, and integrin signaling. Moreover, genes harboring ultrarare variants showed substantial overlap with genes previously implicated in NSCPO, suggesting that ultrarare variants affected key biological pathways underlying NSCPO pathogenesis [99].

##### 3.2.2.5. Elucidation of Genetic Heterogeneity

Multiple WES studies have demonstrated significant genetic heterogeneity in NSOC at several levels. Most notably, substantial differences in genetic architecture exist across populations of different ethnic backgrounds (Table 1). A more diverse genetic background highlights the need for more refined multiethnic sequencing datasets to support accurate disease‐risk prediction. Even within the same population, different affected families may harbor distinct pathogenic variants of the same gene. For example, four different pathogenic variants in *ARHGAP29* were identified across four independent families [100]. In addition, multiple polymorphisms in *IRF6* have been reported in Chinese populations, including c.26G > A [101], c.253A > G [102], c.174 + 1delG [103], c.961C > T [104], and c.1386del [105]. Furthermore, clinical subtypes of NSOC, particularly between NSCL/P and NSCPO, exhibit limited overlap in their polygenic backgrounds. Genetic analyses based on mixed subtype cohorts may, therefore, obscure subtype‐specific association signals. Several WES studies focusing on NSCPO have identified risk variants in genes such as *PARD3* [50] and *LRP6* [106], providing valuable evidence for the subtype‐specific genetic counseling. Existing WES evidence indicates that *GRHL3* may function as a promising causative gene underlying NSCPO. In 2017, Hoebel et al. identified a new variant in *GRHL3* via WES in multiple affected NSCPO families in Central Europe [107], but its role in the etiology of NSCPO in the Chinese population had remained unclear. To fill this gap, Chinese researchers performed WES on a Chinese NSCPO patient, identified a rare missense mutation in *GRHL3* (p.Arg391His), and verified its pathogenicity [108]. Functional assays indicated that p.Arg391His impairs the transcriptional activity of *GRHL3* and that there is a significant difference in nuclear localization patterns between the mutant and wild‐type proteins. The study was the first to clarify the critical pathogenic role of *GRHL3* mutations in the Chinese population, facilitating the understanding of the genetic architecture of NSCPO [108].

**TABLE 1:** Genes and variants implicated in NSOC through WES studies.

Year	Clinical subtype	Population	Sample size	Study design	Genes identified and corresponding variants	PMID
2025	NSOC	Chinese	123 pedigrees	Pedigree	*SCLT1* (c.1908 + 1G > A), *TBC1D32* (c.590_592del), etc.	40147726
2025	NSOC	Chinese	1 pedigree	Pedigree	*IRF6* (c.174 + 1delG)	39679673
2025	NSOC	European and Brazilian	224 pedigrees	Pedigree	*ARHGAP29* (c.2T > C, c.1112delA, c.1939C > T, c.3170delA)	39506048
2025	NSOC	Chinese	214/102 (case/control)	Case–control	*BOC* (c.1219C > T, c.1306G > A, c.3052G > A)	40464334
2025	NSOC	Brazilian	18 pedigrees	Pedigree	*NOTCH1* (c.6898G > A), *KMT2D* (c.3935G > T), *BMP6* (c.1154G > A), *FAM193A* (c.2401G > A), etc.	39855980
2025	NSCL/P	Kuwaiti	20 pedigrees	Pedigree	*SH3PXD2A* (c.242T > C), *DUOX1* (c.4358A > G), *MSX2* (c.371C > T)	41041957
2025	NSOC	Indonesian	6 patients	Case series	*MMACHC* (rs371937044), *SOS1* (rs190222208), *TULP4* (rs199583035), *MTHFD1L* (rs143492706)	37876262
2025	NSOC	Chinese	10 pedigrees	Pedigree	*NRSN2* (p.W57fs)	41624910
2024	NSCL/P	Iranian	1 pedigree	Pedigree	*PDGFC* (c.546dupA)	38432532
2024	NSOC	Chinese	144/102 (case/control)	Case–control	*PTCH2* (c.311T > C, c.392C > G, c.1670G > A, c.2780T > G, c.2933T > A)	38360123
2024	NSOC	Chinese	30 pedigrees	Pedigree	*ECPAS* (c.1931C > G)	38695759
2024	NSOC	Chinese	1 pedigree	Pedigree	*IRF6* (c.1386del)	39114717
2024	NSCL/P	Brazilian	—	Pedigree	*AFDN* (p.Arg669Gln, p.Arg1555His, etc.)	36384317
2023	NSOC	Chinese	107 pedigrees	Pedigree	*CHD7* (c.4033C > T)*, KMT2D* (c.15673C > T), etc.	37745857
2023	NSCPO	Italian and Iranian	35/38 (case/control)	Case–control	*COL2A1* (p.Pro643His)*, GLI3* (p.Gly727Arg)	36830605
2023	NSCL/P	Chinese	1 pedigree	Pedigree	*PDGFC* (c.93G > T)	37779880
2023	NSCL/P	Madagascar	26 pedigrees	Pedigree	*WNT5B* (c.263G > T), *GPC4* (c.965C > G), *MSX1* (c.251A > T), *WDR11* (c.3169A > G), *PHGDH* (c.932C > T), etc.	36980938
2023	NSCPO	Chinese	1 patient	Case	*GRHL3* (p.Arg391His)	35189007
2023	NSCL/P	Chinese	30/‐(case/control)	Case–control	*LAMA5* (c.8251C > T)	36322204
2022	NSCL/P	Chinese	2 pedigrees	Pedigree	*ARHGAP29* (c.1920 + 1G > A)	32698641
2022	NSCPO	Chinese	1 pedigree	Pedigree	*PARD3* (c.1012dupG)	35789100
2022	NSCL/P	Chinese	22 pedigrees	Pedigree	*HMCN2, ANKRD36C, ADGRL2, DIPK2A,* etc*.* (Variants not mentioned)	35701113
2021	NSOC	Chinese	1 pedigree	Pedigree	*PTCH1* (c.2833C > T)	34291140
2021	NSCLP	Chinese	1 pedigree	Pedigree	*IRF6* (c.253A > G)	33423258
2021	NSCL/P	Chinese	1 pedigree	Pedigree	*TP63* (c.1324C > T)	33622322
2021	NSCL/P	Chinese	1 pedigree	Pedigree	*IRF6* (c.961C > T)	33136784
2021	NSCPO	Brazilian	30/30 (case/control)	Case–control	*LRP6* (rs34815107), *MTHFR* (rs45496998), *MTR* (rs113277607), *CDH1* (rs33969373), etc.	34307341
2021	NSCL/P	Chinese	1 pedigree	Pedigree	*PAX3* (c.92C > G)	34918979
2020	NSCL/P	Chinese	1 pedigree	Pedigree	*ARHGAP29* (c.2615C > T)	33150183
2020	NSCL/P	Honduran	1 pedigree	Pedigree	*IRF6* (c.921C > T)	32784565
2020	NSOC	Saudi Arabian	1 pedigree	Pedigree	*NRP1 (*rs35320960), *RPL27A* (rs199996172)	33121284
2019	NSCL/P	Chinese	1 pedigree	Pedigree	*GLI2* (c.2684C > T, c.4350G > T, c.4622C > A)	31386309
2019	NSCL/P	Chinese	1 pedigree	Pedigree	*CDH1* (c.468G > C)	31638429
2019	NSOC	Caucasian	7 pedigrees	Pedigree	*FGFR1* (c.1809_1810insTC), *DLG1* (c.832C > G)	31652620
2018	NSOC	Chinese	1 pedigree	Pedigree	*PTCH1* (c.1175C > T)	29908092
2018	NSCL/P	Chinese	1 pedigree	Pedigree	*IRF6* (c.26G > A)	30053123
2018	NSCL/P	Multiethnic	72 pedigrees	Pedigree	*CTNND1* (p.Gln19Glu), *PLEKHA7* (p.Gly544Asp), *PLEKHA5* (p.Tyr590Cys), *ESRP2* (p.Arg315His)	29805042
2018	NSOC	—	46 pedigrees	Pedigree	*TP63* (c.819_820dupCC), *TBX1* (c.805C > T), *LRP6* (c.3373C > T), *GRHL3* (c.1171C > T)	29500247
2017	NSCPO	European	8 pedigrees	Pedigree	*ACACB* (T > G), *PTPRS* (G > A), *MIB1* (G > A), *GRHL3* (A > G), *CREBBP* (G > C)	28767323
2017	NSCL/P	Honduran	27 pedigrees	Pedigree	*ADH7, AHR, CRYZ* (3 CNCs detected corresponding to these 3 genes)	28748635
2017	NSCPO	European	1 pedigree	Pedigree	*ARHGAP29* (c.1654T > C)	28029220
2016	NSOC	Colombian	7 pedigrees	Pedigree	*MSX1* (c.778C > A), *MYH3* (c.3869G > A), etc.	27456059
2016	NSCL/P	European	1 pedigree	Pedigree	*MYO5B* (rs183559995), *LOXHD1* (rs1450425), *SKA1* (rs6507992), *SMAD7* (rs78950893), etc.	27242896
2016	NSCLP	Honduran	27 pedigrees	Pedigree	*ACSS2* (p.Val496Ala), *PHYH* (p.Arg245Gln)	27229527
2015	NSOC	European	1 pedigree	Pedigree	*DLX4* (c.546_546delG)	25954033
2015	NSCL/P	Chinese	8 patients	Case series	*CENPJ* (c.61T > C), *HEATR8* (c.1526T > C), *REG3A* (c.149T > G), *AQP7* (c.538C > T)	26449438
2014	NSOC	Indian	55 pedigrees	Pedigree	*CDH1* (G > T)	24793288

*Note:* NSCL/P: nonsyndromic cleft lip with or without cleft palate; —: not mentioned.

Abbreviations: CNCs, copy number changes; NSCLP, nonsyndromic cleft lip and palate; NSCPO, nonsyndromic cleft palate only; NSOC, nonsyndromic orofacial clefts.

Overall, WES has substantially advanced the discovery of novel disease‐associated genes in NSOC and significantly enhanced the ability to elucidate its etiology. However, WES has limited capacity to detect non‐coding variants and structural variants, such as large CNVs and SVs, and may also miss pathogenic variants in the regulatory regions [109].

### 3.3. WGS: Detection of Noncoding Variants and SVs

Non‐coding regions of the genome, including promoters, enhancers, and other regulatory elements, as well as large‐scale SVs play critical roles in the pathogenesis of many complex diseases [110–112]. WGS is not restricted to exonic or predefined functional regions, while it provides an unbiased and comprehensive assessment of the entire genome. WGS enables the detection of a broad spectrum of genetic variation, including noncoding variants, structural rearrangements, CNVs, and microsatellite instability, thereby serving as an important complement to WES or surpassing the capabilities of WES [109]. Although WGS produces greater data volume and is associated with analytical complexity and higher cost compared with WES, ongoing technological advances and continued reductions in sequencing costs are expected to facilitate its wider adoption. Consequently, WGS is likely to be a powerful tool for both basic research and translational studies of genetic diseases.

#### 3.3.1. Major Findings From WGS Studies in NSOC

By interrogating genomic regions that are largely inaccessible to WES, WGS enables the comprehensive detection of both common and rare variants associated with NSOC risk (Table 2). Alvarado et al. applied WGS in a three‐generation extended pedigree combined with polygenic risk score (PRS) analysis, which identified a rare missense mutation in *PDGFRA* (c.2740C > T). They demonstrated that the phenotypic effect of this variant was modified by additional common NSCL/P risk variants [113]. In another study, Zhong et al. identified rs2385404 and rs2385405 in *RESP18* as novel risk loci for NSCLP in a five‐member family from the southern Han Chinese population [114]. These mutations directly lead to abnormalities in the structure and function of the *RESP18* protein, which regulates the growth of cytoskeleton and affects organ development through post‐transcriptional O‐glycosylation under normal conditions. Notably, the genotypic dose effect of homozygous mutations significantly exacerbates the severity of NSCLP [114]. WGS also expanded understanding of the contribution of DNMs to NSOC. In a study of 130 African NSCL/P families, researchers identified 162 protein‐altering DNMs in genes including *ARHGAP10* [115]. An analysis of 756 families from European, Colombian, and Taiwanese populations revealed an enrichment of loss‐of‐function DNMs in genes highly expressed in craniofacial tissues [116].

**TABLE 2:** Genes and variants implicated in NSOC through WGS studies.

Year	Clinical subtype	Population	Sample size	Study design	Identified genes and corresponding variants	PMID
2024	NSCLP	Chinese	1 pedigree	Pedigree	*RESP18* (rs2385404, rs2385405)	38970244
2024	NSCL/P	African	130 pedigrees	Pedigree	*AFDN* (p.Lys12Arg), *ADAM23* (p.Met1ext‐28), *ITGA6* (p.Pro67Leu)	36384317
2022	NSCL/P	European	1 pedigree	Pedigree	*PDGFRA* (c.2740C > T)	35147171
2022	NSCPO	European	30 patients	Case series	*TBX22* (c.821_824del), *COL2A1* (c.2301 + 1G > A), *FBN1* (c.7754T > C), *PCGF2* (c.930dup), *KMT2D* (c.11905C > T)	35281813
2022	NSCL/P	African	130 pedigrees	Pedigree	*ACAN* (c.4321A > G), *DHRS3* (c.110C > T), *DLX6* (c.490G > C), *ACTL6A* (c.2T > G), *ARHGAP10* (c.784C > T), etc.	35817949
2020	NSOC	European and Colombian	580 pedigrees	Pedigree	*PDE9A* (rs2839575), *PAX7* (rs78998514), etc.	31848685
2020	NSOC	European, Colombian and Taiwanese	756 pedigrees	Pedigree	*TFAP2A* (p.Glu104^∗^), *IRF6* (p.Gly376Val), etc.	32574564

*Note:* NSCL/P: nonsyndromic cleft lip with or without cleft palate.

Abbreviations: NSCLP, nonsyndromic cleft lip and palate; NSCPO, nonsyndromic cleft palate only; NSOC, nonsyndromic orofacial clefts.

^∗^here indicates a nonsense mutation which results in a premature termination codon occurrence.

In terms of genetic heterogeneity, Mukhopadhyay et al. conducted WGS in European and Colombian families based on a multicenter study on cleft lip and palate, in which identified a population‐specific risk locus, rs2839575 (*PDE9A*), on chromosome 21 in Colombian pedigrees [117]. *PDE9A* has been shown to overlap a superenhancer region for craniofacial development identified from histone profiling in early human craniofacial development [118]. Notably, this variant was not detected in large NSOC cohorts from other Latin American populations [117]. Another WGS study in 30 NSCPO patients identified potentially pathogenic variants in *TBX22*, *COL2A1*, *FBN1*, *PCGF2*, and *KMT2D*, providing a genetic basis for precision genetic counseling and disease prevention strategies [119].

Integration of WGS with animal models has enabled deeper exploration of regulatory‐region mutations. WGS analysis of the Grhl2Axd mouse line, which exhibits a cleft lip and palate phenotype, identified an approximately 4kb long terminal repeat retrotransposon insertion disrupting a noncoding regulatory region of *Grhl2*, leading to overexpression of the gene [120].

#### 3.3.2. Sequencing‐Based Association Studies

Traditional GWASs rely primarily on SNP array technologies and large‐scale case–control statistical analyses to identify disease‐associated genetic variants [121]. However, SNP array‐based GWASs have inherent limitations in detecting low‐frequency variants. Studies have shown that even when using high‐density genotyping arrays, approximately 39.2%–56.6% of low‐frequency SNPs remain untagged, which was substantially higher than the proportion of untagged common SNPs [122]. Moreover, the commercial Illumina 1M array covers less than 10% of low‐frequency variants present in the study samples. In contrast, NGS enables comprehensive capture of the full allele frequency spectrum and provides significantly greater statistical power for association testing compared with array‐based approaches [122]. In future NSOC research, promoting a methodological transition from “Array‐based GWAS” to “Sequencing‐based Association Studies” is expected to uncover additional low‐frequency and rare pathogenic variants, thus further refining the genetic architecture of the disease.

#### 3.3.3. Detection of CNVs

Methods for detecting CNVs have advanced rapidly with the emergence of NGS technologies, which have substantially enhanced the investigation of complex genomic variants due to high throughput, reproducibility, and accuracy [123]. Although WES can provide read counts for CNV inference, it lacks sufficient resolution for reliable detection of microduplications and microdeletions. WGS, compared with WES, introduces less bias and genomic noise, which helps overcome these limitations and makes CNV detection less challenging [124]. Using 22q11.2 deletion syndrome, a congenital disorder frequently accompanied by cleft palate, as an example, Guo et al. applied WGS and successfully confirmed a 3Mb de novo deletion in the 22q11.2 region of the proband through read‐depth analysis. By integrating WGS data with BAC clone‐based variation maps, the authors preliminarily localized the deletion breakpoint interval and inferred that the breakpoint most likely resides near or within the BCRP module [125]. This finding highlights the considerable potential of WGS for providing precise and detailed CNV characterization.

## 4. Third‐Generation Sequencing (TGS) and Precise Characterization of Complex Structural Variants

### 4.1. Technical Characteristics

Although NGS offers a clear advantage in throughput, its relatively short read lengths require complex sequence assembly and rely heavily on PCR amplification during library preparation. The emergence of TGS has largely addressed these limitations. Also known as single‐molecule sequencing, TGS is characterized by real‐time sequencing of individual DNA molecules and long read lengths. Current TGS platforms mainly include PacBio single‐molecule real‐time sequencing (SMRT), nanopore sequencing, and Helicoscope single‐molecule sequencing [126]. Taking SMRT sequencing as an example, this technology utilizes zero‐mode waveguides (ZMWs) to enable independent sequencing of single DNA molecules, effectively minimizing molecular interference. Compared with NGS, TGS offers clear advantages in high‐contiguity de novo genome assembly, detection of complex SVs (including CNVs, inversions, mobile element insertions, and ultralong tandem repeats), full‐length transcript and isoform identification, and base‐modification detection [127]. For instance, in cancer research, Patel et al. developed the Amplification of Breakpoints (AmBre) strategy, which employs a simulated annealing algorithm to design primers for breakpoint‐specific PCR amplification, followed by PacBio sequencing to achieve precise identification and validation of tumor structural‐variant breakpoints. Using this approach, deletion breakpoints in *CDKN2A* were successfully characterized in A549 cells and bidirectional translocation breakpoints involving *RUNX1*–*RUNX1T1* were identified in the Kasumi‐1 cell line, demonstrating excellent sensitivity for capturing low‐abundance structural‐variant breakpoints [128]. These findings highlight the ability of TGS to overcome the inherent limitations of short‐read sequencing in resolving complex structural variation.

### 4.2. Reasons Underlying the Limited Application of TGS in NSOC Research and Future Perspectives

Despite the significant transformative technological advantages of TGS, its application in NSOC research remains exceedingly limited. Currently, the common understanding is that the cumulative effect of multiple single nucleotide variations, each with relatively mild effects, is the primary genetic factor in NSOC, whereas highly pathogenic large structural variations are mainly linked to the syndromic form of orofacial cleft. Employing TGS to screen for complex structural variations in NSOC does not appear to be a cost‐effective strategy. Additionally, compared with established NGS platforms, the costs and technical requirements (especially DNA quality requirements) associated with TGS applications are still relatively high, thereby restricting its use in large‐scale case–control and family‐based studies. Future investigations should still focus on utilizing TGS to detect complex structural variations in NSOC, as these variations may represent important components of “missing heritability.” Furthermore, in the field of epigenetics, given the capability of TGS to identify base modifications and perform full‐length transcript isomer analysis, it is expected to enhance our understanding of the DNA methylation profiles and the disease‐related RNA splicing mechanisms.

## 5. Sequencing Technologies Drive the Elucidation of Pathogenic Mechanisms and Functional Insights in NSOC

### 5.1. NGS Technologies for Epigenomic Studies in NSOC

Epigenomics refers to the use of genome‐wide measurement to characterize the global patterns of DNA modifications (particularly cytosine methylation) and chromatin states in eukaryotes [129]. This field is one of the primary areas where NGS technology is extensively utilized. A key advantage of the NGS platform is its capacity to analyze the epigenome without bias, thus overcoming the inherent limitations of traditional chip technology regarding detection scope.

DNA methylation is a fundamental mechanism of epigenetics. Various studies have demonstrated that specific abnormal methylation profiles are closely associated with susceptibility to NSOC, utilizing samples from blood, saliva, lip and palate tissues, and mouse models [130–132]. The integration of bisulfite sequencing with high‐throughput sequencing enables high‐quality analysis of DNA methylation profiling, allowing for precise assessment of the methylation status across the entire genome or within key genomic regions [133]. Zhang et al. conducted whole‐genome bisulfite sequencing (WGBS) in eight injured and five self‐normal lip tissue samples from children with NSCLP and identified 2711 differentially methylated regions (DMRs) [134]. Researchers further observed hypomethylation of the *GLI2* gene promoter, along with elevated mRNA expression levels in the injured lip tissue. Young et al. conducted WGBS on six monozygotic twin pairs discordant for NSCLP, successfully identifying differentially methylated positions (DMPs) and DMRs in genes such as *MAFB* and *ZEB2* [135]. Moreover, their study revealed common differential methylation of genes associated with the Hippo signaling pathway, suggesting a link between the mechanosensory pathway and the etiology of NSCLP. Abnormal histone modifications may serve as a bridge in gene‐environment interactions, thereby elevating the risk of NSOC [136, 137]. Researchers collected craniofacial samples from human embryos at four distinct Carnegie stages (CS13, CS14, CS15, and CS17) and detected multiple histone modifications using chromatin immunoprecipitation followed by NGS (ChIP‐seq) [118]. To assess chromatin states, ATAC‐seq has emerged as a robust method for characterizing chromatin accessibility. This method can effectively map the open and closed chromatin conformations in the genome with minimal cell volume and within a short timeframe [138]. In recent years, massively parallel capability of genome sequencing have facilitated the application of 3C, 4C, 5C, and Hi‐C technologies to analyze high‐resolution 3D genome folding [139]. This advancement has significantly contributed to the development of 3D epigenomics and the understanding of higher‐order chromatin structures. The integrated use of multiple sequencing technologies capitalizes on the strengths of each method, resulting in a more comprehensive epigenetic map and establishing a prevalent research strategy [140]. For instance, one study constructed a dataset of the oral epithelial integrated epigenome and 3D chromatin conformation by employing RNA‐seq, ATAC‐seq, H3K27ac ChIP‐seq, and digestion‐ligation‐only Hi‐C (DLO) on human oral epithelial cells [141]. The approach accurately identified potential risk functional variations located within active cis‐regulatory elements. These risk variants disrupt palatal development through interference with epithelial‐specific enhancer activity, transcription factor binding, and target gene expression [141].

In summary, advancements in epigenomic sequencing technologies are driving a more profound investigation into epigenetic regulatory mechanisms, such as uncovering DNA methylation, histone modification, interactions among regulatory elements, and 3D chromatin structure. Unraveling the molecular mechanisms underlying epigenome function relies fundamentally on robust, reproducible, and streamlined technologies capable of producing data that can be directly integrated into existing omics databases [142]. These sequencing technologies can be used in both human control samples versus patient‐derived samples, as well as with animal models (mutants as compared with the wildtype) [143]. When coupled with single‐cell methodologies which will be discussed in detail in the next section, these investigations will further enhance our comprehension of the epigenetic landscape of NSOC, offering potential biomarkers and novel genetic approaches for early disease prevention.

### 5.2. Single‐Cell Sequencing

#### 5.2.1. Technical Characteristics

The sequencing approaches mentioned in Sections 2, 3 and 4 have primarily focused on the identification of susceptibility genes and genetic variants associated with NSOC. However, there is a limited study examining how these variants lead to the development of cleft lip and palate within specific cell types and spatiotemporal contexts. In recent years, the rapid advancement of NGS technologies has not only provided abundant genetic data resources for NSOC research but has also enabled the refinement of sequencing methodologies toward greater precision and higher resolution through high‐throughput platforms. Currently, NGS‐based multiomics studies are increasingly focused on the characterization of the cellular features at the single‐cell level. In contrast to traditional bulk sequencing, which only provides averaged signals across heterogeneous samples, single‐cell sequencing offers a powerful tool for dissecting cellular and molecular landscapes at single‐cell resolution [144, 145]. Among these technologies, single‐cell RNA sequencing (scRNA‐seq) enables the reconstruction of differentiation trajectories of cellular subpopulations during various stages of craniofacial development, thereby facilitating integrative analyses linking molecular characteristics with cell lineages [146]. In parallel, single‐cell assay for transposase‐accessible chromatin sequencing (scATAC‐seq) profiles cell‐type‐specific chromatin accessibility landscapes, providing critical guidance for the identification of active regulatory elements and the inference of DNA–protein binding sites [138, 147].

#### 5.2.2. Applications of scRNA‐Seq in Cleft Lip and Palate Research

As an ideal model organism, the mouse is the primary system for applying scRNA‐seq to analyze developmental patterns of the lip and palate. Using scRNA‐seq, Cai and Yin successfully identified distinct subpopulations of mesenchymal and ectodermal cells involved in palatal fusion, characterized their unique transcriptomic profiles, and reconstructed their differentiation trajectories. Through further integrating scATAC‐seq data, the authors constructed dynamic intercellular communication networks and predicted upstream transcriptional regulators of the key signaling pathway genes [148].

Huang et al. analyzed single‐cell profiles of mouse palatal tissues from embryonic day E10.5–E16.5 and identified four subpopulations of periderm cells. Their findings demonstrated that epithelial–mesenchymal transition, apoptosis, and cell migration jointly contribute to periderm regression within the medial epithelial seam [149]. Ozekin et al. investigated cellular heterogeneity in the anterior palate at E13.5, which identified a distinct mesenchymal cell population with specialized functions based on differential ligand‐receptor expression patterns [150]. Additionally, Sun et al. showed that Ezh2‐mediated histone H3K27 methylation suppressed expression of the cell‐cycle regulator *Cdkn1a,* promoting the proliferation of palatal shelf epithelial cells [151].

In addition, several studies indicated that integrating scRNA‐seq with complementary analytical strategies can yield deeper insights into the pathogenic mechanisms underlying cleft lip and palate. For example, Itai et al. demonstrated that combining pathway analysis with scRNA‐seq data facilitated more precise contextual interpretation of gene function during cleft palate development [152]. Moreover, incorporating scRNA‐seq into comparative designs between disease models and healthy controls enabled the identification of aberrant gene regulatory networks from a novel perspective. In a comparative scRNA‐seq analysis of *Kcnj2* potassium channel homozygous knockout mice (cleft palate model) and wild‐type controls at E13.5, *Kcnj2* deficiency was shown to alter calcium‐induced transcription factor–related regulatory networks as well as disrupt gene transcription in *Kcnj2*‐nonexpressing cells, leading to the impaired BMP signaling [153]. Wang et al. further reported a significantly increased proportion of myeloid cells in palatal tissues from a retinoic acid‐induced cleft palate mouse model, with particularly pronounced increases in M1 macrophages and monocytes [154]. Finally, using scRNA‐seq data in combination with the single‐cell disease relevance score (scDRS) and differential gene expression analyses, Siewert et al. identified two major cell subtypes closely associated with the occurrence of NSCL/P [155].

At present, single‐cell sequencing technologies still face several practical challenges. Although single‐cell sequencing enables efficient identification of novel cell populations, the lack of standardized frameworks for annotating and reporting cell subpopulation characteristics, together with the tendency of many studies to focus primarily on cell‐type identification without consensus on the downstream application, has substantially limited the data integration, reproducibility, and clinical translation [156]. Future efforts should prioritize the establishment of standardized reporting guidelines for single‐cell sequencing results and the development of transparent, open data‐sharing platforms to enhance cross‐study comparability.

### 5.3. Spatial Omics

#### 5.3.1. Technical Characteristics

Although single‐cell sequencing has largely overcome the limitations of traditional bulk analyses methods, it faces a new challenge: the loss of spatial and microenvironmental information caused by tissue dissociation during sample processing. The emergence of spatial omics technologies has made it possible to study the proteins and transcripts in situ while preserving anatomical and histological context [157]. Notably, recent integration of single‐cell sequencing with spatial omics has led to the development of single‐cell spatial omics technologies, which combine single‐cell resolution with spatial localization information [158]. By combining the complementary strengths of both approaches, single‐cell spatial omics has become a powerful tool for dissecting the spatiotemporal developmental trajectories of tissues and organs. Among these, single‐cell spatial transcriptomics (SCS) has shown the most rapid progresses.

#### 5.3.2. Applications of Spatial Omics in Orofacial Cleft Research

In the NSOC research, Wang et al. performed scRNA‐seq and spatial enhanced resolution omics sequencing (Stereo‐seq) on mouse embryonic palatal tissues to identify distinct cellular subpopulations and characterize their spatial distribution patterns [159]. By integrating two sequencing data, the authors annotated mesenchymal and epithelial cell populations during palatal development and reconstructed intercellular communication networks. Their results demonstrated that at E14.5 period, Tgfβ3 and Pthlh signaling were specifically enriched in the midline epithelial seam, suggesting a potential role in epithelial–mesenchymal transition [159]. These findings provide novel insights into the intercellular signaling mechanisms during palatogenesis. Pina et al. applied spatially resolved RNA sequencing to the secondary palate and identified transcriptional program shifts, marking the initiation of osteogenesis between E14.5 and E15.5, defining the spatial expression patterns of key osteogenic marker genes. This study offers a new perspective for developing cleft palate–specific diagnostic biomarkers [160].

While the mouse models offer valuable insights for spatial omics research, disparities exist between mice and humans in terms of the genome, developmental processes, the exposed environment, and tissue structure. These discrepancies pose challenges in extrapolating omics findings to the human NSOC mechanism. Notably, most omics studies utilize genetically homogeneous inbred mouse strains or single‐gene knockout models for cleft lip and palate. In contrast, human NSOC is a complex polygenic disorder featuring significant genetic heterogeneity and intricate gene–environment interactions. Consequently, molecular pathogenic mechanisms identified in mouse models have limited applicability to the majority of sporadic human NSOC cases. Moreover, stringent ethical constraints on sampling human embryonic craniofacial tissue, particularly during critical developmental stages, have resulted in a scarcity of human tissue validation for spatial omics discoveries.

The application of spatial omics technologies also faces other limitations. First, the high cost of commercial spatial omics platforms restricts their large‐scale application, while the in‐house methods often suffer from lower throughput and insufficient standardization [161]. Second, at the technical level, spatial omics approaches impose stringent requirements on sample quality and processing, complex data‐analysis pipelines, and substantial computational resources, and face persistent challenges in accurate cell segmentation. In addition, the large volume of spatial omics data poses significant difficulties for data storage and management [162]. Finally, the field of spatial omics urgently requires more robust standardization frameworks to improve cross‐study comparability.

### 5.4. Multiomics

#### 5.4.1. Technical Characteristics

Single‐cell multiomics technologies integrate multiple single‐modality omics approaches, including transcriptomics, genomics, epigenomics, epitranscriptomics, proteomics, and metabolomics, which enable multidimensional characterization of cellular states and activities. These technologies drive a transformative shift in the molecular and cellular biology research [163]. Their core features include methodological diversity, high‐throughput data generation, integrated analytical workflows, and application‐specific design [163].

Compared with single‐omics approaches, multiomics technologies present with substantial advantages. By overcoming the limitations of individual omics methods, which often provide correlative evidence without fully elucidating molecular functions and interactions, multiomics integration enables complementary strengths to be leveraged for the interpretation of the biological processes. These approaches promote early disease risk prediction, precise molecular subtyping and efficient biomarker discovery, and support large‐scale medical cohort studies. Multiomics technologies will provide critical methodological support for the clinical translation of precision medicine [164].

#### 5.4.2. Research Advances in the Integration of Multiomics Data in Orofacial Cleft

Yan et al. performed sequencing analyses of the secondary palate in mouse embryos and reconstructed five differentiation trajectories of neural crest cells [165]. By integrating open chromatin accessibility signals with gene expression dynamics, key transcription factors governing lineage specification were identified. Among these, *SHOX2* and *MEOX2* were shown to play critical roles in regulating anterior and posterior palatal development, respectively. These findings indicate the dynamic transcriptional changes during palatogenesis and provide a foundation for subsequent studies on cleft palate. Homozygous deletion of *Pax9* (Pax9^−/−^) mice model results in a cleft palate phenotype [40]. Using single‐cell spatial multiomics approaches, researchers further revealed a clear association between *Pax9*‐positive cells and osteoblast populations within the developing palate. Deletion of *Pax9* led to abnormal spatial distribution of osteogenic regions and disrupted normal osteogenic differentiation of mesenchymal cells [166]. Collectively, these findings demonstrate that integrating multiomics datasets and constructing developmental or pathogenic multiomics models can facilitate more refined identification of key regulatory elements, thereby providing important insights into the etiology of cleft lip and palate and informing strategies for precision prevention and intervention.

Similar to the two technologies mentioned above, the application of multiomics approaches continues to face several limitations, including substantial heterogeneity in analytical standards, high costs and operational complexity, limited data reusability and barriers to interoperability, and cross‐base integration of research findings [167]. In the future, the multiomics field should move toward greater open‐access and data‐sharing frameworks to enhance reproducibility. In parallel, the integration of multi‐omics data with artificial intelligence and machine learning models holds promise for overcoming the constraints of conventional analytical approaches and advancing deeper biological insight [168].

Utilizing omics techniques, researchers have delved into the etiology of NSOC from various perspectives, thereby establishing a theoretical foundation for further exploration of its molecular mechanisms. Table 3 provides a summary of the correlations between certain susceptibility genes discussed in Sections 2 and 3 and the cell subpopulations identified through omics analyses. This highlights that omics technology has substantially improved our understanding of the susceptibility genes identified in the conventional sequencing era by elucidating their functions within specific spatiotemporal contexts and cell subpopulations.

**TABLE 3:** Correlation between some of the susceptibility genes detected by sequencing technologies and cell subgroups identified by omics analyses.

Marker genes of cell subgroups	Sites of gene expression and developmental stages	Cell types	Related biological patterns	PMID
*Vax1*	Lambdoidal junction tissue derived from mouse embryos (E10.5–E12.5)	Ectodermal cells	Involvement in the developmental regulation of the upper lip and primary palate	39392189
*Irf6, Grhl3, Arhgap29*	Lambdoidal junction tissue derived from mouse embryos (E10.5–E12.5)	Ectodermal cells	The main receiving subgroup of NOTCH signaling; prevention of pathological epithelial adhesion during the embryonic period	39392189
*Pax9*	Palatal tissue of mouse embryos (E10.5–E16.5)	Mesenchymal cells, periderm cells in the trajectory of fusion	Regulation of cell proliferation and lineage differentiation, mediation of key signaling pathways such as Wnt and Shh, and promotion of palatal shelf growth, tissue differentiation, and morphogenesis	40037804
*Pax9*	Palatal tissue of mouse embryos (E13.5–E15.5)	*PAX9*‐positive mesenchymal cells	A key transcription factor in palatogenesis that governs the growth, elevation, and fusion of palate	37709732
*Klf4*	The oral‐side surface of the palatal shelf not in contact with the contralateral palatal shelf (E16.5)	Keratinized periderm cell II	Completion of terminal keratinization and stratum corneum formation and construction of the mature epithelial barrier	40037804
*Msx1*	Anterior palate of the mouse (E13.5)	Mesenchymal cells in the central palatal region	An essential molecule for ensuring the normal transition of the mouse anterior palate from the vertical proliferation phase to the palatal shelf elevation phase.	36734036
*Bmp4, Gli3, Pbx1*	Lambdoidal junction tissue derived from mouse embryos (E11.5)	Mesenchymal cells and ectodermal cells	Mediation of epithelial–mesenchymal interaction and regulation of cell proliferation, differentiation, adhesion, and migration	38807368
*Tbx1*	Lambdoidal junction tissue derived from mouse embryos (E11.5)	Endothelial cells	Mediation of tissue morphogenesis and differentiation	38807368
*ZFP36L2*	Human craniofacial epithelial tissue (CS12–CS16)	HAND2‐positive pharyngeal arch mesenchymal cells	Involvement in the development of hard and soft tissues including the roof of the mouth, tongue, craniofacial muscles, and blood vessels	39489835
*Col2a1*	Middle palate of the embryonic mouse (E12.5–E16.5)	Chondrogenic cells	Regulation of chondrogenesis during palatal development	38619065
*Runx2*	Secondary palate of mouse embryos (E13.5–E15.5)	Preosteoblasts	Driving directed differentiation towards mature osteoblasts	38910391

## 6. Summary and Future Perspectives

Over the past several decades, advances in sequencing technologies have driven transformative developments in the genetic study of NSOC, enabling a transition from targeted single‐gene analyses to genome‐wide investigations of genetic susceptibility, and from variant identification to in‐depth interpretation of pathogenic mechanisms. Sanger sequencing laid the foundation for identifying core susceptibility genes associated with NSOC, while NGS has substantially expanded the scope of variant discovery, allowing effective detection of rare variants and noncoding variants. TGS, as an emergency strategy, is available for comprehensive investigation of complex SVs. In parallel, epigenomics, single‐cell sequencing, spatial omics, and multi‐omics integration have further established links between genetic factors and spatiotemporal cellular phenotypes.

Nevertheless, it is imperative to acknowledge the substantial challenges encountered by these sequencing technologies, including heavy expenses, the absence of standardized analytical procedures and agreement, and barriers to clinical application. Another common and important understanding is that the poor extrapolation capability resulting from genetic heterogeneity across populations poses a major obstacle to the application of sequencing technology in genetic research. Forcibly applying SNPs identified in one population to others may introduce significant epidemiological bias. We hereby recognize a limitation inherent to our work. The studies included in this narrative review are heavily weighted toward those conducted in Chinese populations. Despite providing certain relevant insights, it fails to comprehensively capture the genetic diversity present in other populations.

Future efforts should prioritize technological optimization, strengthened data‐sharing infrastructures, and deeper integration of multiplatform and multiomics approaches. Specific emphasis should be placed on the development of population‐specific genetic reference databases and the identification of subtype‐specific etiological mechanisms, alongside efforts to bridge the gap between basic research and clinical application. Recent studies indicate that PRSs are expected to become a powerful tool for translating genetic advancements into clinical practice. Most risk variants in complex diseases exhibit modest effect sizes [169], leading to the limited predictive value of individual SNPs. PRS evaluates the polygenic background and genetic risk by aggregating the effect sizes of risk and protective alleles carried by individuals [170], demonstrating effective risk stratification in diseases such as cancer [171, 172] and cardiovascular diseases [173]. In the realm of NSOC, research on PRS diagnostic prediction models remains limited and is still in its early stages. Although several PRS models have exhibited remarkable performance [174–177], they encounter several major limitations. The challenges include the impact of genetic heterogeneity across populations, the inability of included SNPs to comprehensively represent the genetic background, and the absence of standardized guidelines and consensus in model development. It is noteworthy that the PRS models developed in NSOC are predominantly derived from conventional SNP genotyping arrays used for GWAS. While SNP arrays and WGS exhibit similar efficacy in identifying common variants, utilizing WGS for genotyping enables more powerful detection of signals with low minor allele frequency [178]. This advantage enables PRS to capture a more comprehensive profile of genetic susceptibility. However, despite the technical feasibility of replacing existing methods with WGS, its cost remains a significant barrier. Particularly, a recent investigation on Type 1 diabetes has demonstrated that employing the Trans‐Omics for Precision Medicine (TOPMed)–imputed array genotypes for PRS computation offers a cost‐effective and efficient alternative to the WGS strategy [179]. In the future, the incorporation of WGS data into the construction of NSOC PRS will necessitate a thorough and careful economic evaluation. For a comprehensive risk assessment of NSOC, the integration of environmental variables and epigenetic markers into the PRS model is also essential, along with exploring the potential role of machine learning algorithms in improving prediction accuracy. Such efforts will provide a robust technical foundation for improving comprehensive birth‐defect prevention strategies, advancing secondary prevention, and promoting the development of precision medicine.

## Funding

No funding was received for this manuscript.

## Ethics Statement

The authors have nothing to report.

## Conflicts of Interest

The authors declare no conflicts of interest.

## Data Availability

Data sharing is not applicable to this article as no datasets were generated or analyzed during the current study.

## References

[1] Mossey P. A. , Little J. , Munger R. G. , Dixon M. J. , and Shaw W. C. , Cleft Lip and Palate, Lancet. (2009) 374, no. 9703, 1773–1785, 10.1016/s0140-6736(09)60695-4, 2-s2.0-70449629175. 19747722

[2] Woodward A. A. , Urbanowicz R. J. , Naj A. C. , and Moore J. H. , Genetic Heterogeneity: Challenges, Impacts, and Methods Through an Associative Lens, Genetic Epidemiology. (2022) 46, no. 8, 555–571, 10.1002/gepi.22497. 35924480 PMC9669229

[3] Selleri L. and Rijli F. M. , Shaping Faces: Genetic and Epigenetic Control of Craniofacial Morphogenesis, Nature Reviews Genetics. (2023) 24, no. 9, 610–626, 10.1038/s41576-023-00594-w. 37095271

[4] Dixon M. J. , Marazita M. L. , Beaty T. H. , and Murray J. C. , Cleft Lip and Palate: Understanding Genetic and Environmental Influences, Nature Reviews Genetics. (2011) 12, no. 3, 167–178, 10.1038/nrg2933, 2-s2.0-79951800135. PMC3086810 21331089

[5] Goodwin S. , McPherson J. D. , and McCombie W. R. , Coming of Age: Ten Years of Next-Generation Sequencing Technologies, Nature Reviews Genetics. (2016) 17, no. 6, 333–351, 10.1038/nrg.2016.49, 2-s2.0-84968903135. PMC10373632 27184599

[6] Shendure J. , Balasubramanian S. , Church G. M. et al., DNA Sequencing at 40: Past, Present and Future, Nature. (2017) 550, no. 7676, 345–353, 10.1038/nature24286, 2-s2.0-85031940779. 29019985

[7] Sanger F. , Nicklen S. , and Coulson A. R. , DNA Sequencing With Chain-Terminating Inhibitors, Proceedings of the National Academy of Sciences of the United States of America. (1977) 74, no. 12, 5463–5467, 10.1073/pnas.74.12.5463, 2-s2.0-0017681196. 271968 PMC431765

[8] Shendure J. and Ji H. , Next-Generation DNA Sequencing, Nature Biotechnology. (2008) 26, no. 10, 1135–1145, 10.1038/nbt1486, 2-s2.0-53649106195. 18846087

[9] Vieira A. R. , Avila J. R. , Daack-Hirsch S. et al., Medical Sequencing of Candidate Genes for Nonsyndromic Cleft Lip and Palate, PLoS Genetics. (2005) 1, no. 6, 10.1371/journal.pgen.0010064. PMC1298935 16327884

[10] Watanabe A. , Akita S. , Tin N. T. et al., A Mutation in RYK is a Genetic Factor for Nonsyndromic Cleft Lip and Palate, The Cleft Palate-Craniofacial Journal. (2006) 43, no. 3, 310–316, 10.1597/04-145.1. 16681403

[11] Koboldt D. C. , Steinberg K. M. , Larson D. E. , Wilson R. K. , and Mardis E. R. , The Next-Generation Sequencing Revolution and Its Impact on Genomics, Cell. (2013) 155, no. 1, 27–38, 10.1016/j.cell.2013.09.006, 2-s2.0-84884826911. 24074859 PMC3969849

[12] Schutte B. C. and Murray J. C. , The Many Faces and Factors of Orofacial Clefts, Human Molecular Genetics. (1999) 8, no. 10, 1853–1859, 10.1093/hmg/8.10.1853, 2-s2.0-0032881718. 10469837

[13] Vieira A. R. , Modesto A. , Meira R. , Barbosa A. R. , Lidral A. C. , and Murray J. C. , Interferon Regulatory Factor 6 (IRF6) and Fibroblast Growth Factor Receptor 1 (FGFR1) Contribute to Human Tooth Agenesis, American Journal of Medical Genetics, Part A. (2007) 143, 538–545, 10.1002/ajmg.a.31620, 2-s2.0-33847368959. PMC2570343 17318851

[14] van den Boogaard M. J. , Dorland M. , Beemer F. A. , and van Amstel H. K. , MSX1 Mutation is Associated With Orofacial Clefting and Tooth Agenesis in Humans, Nature Genetics. (2000) 24, no. 4, 342–343, 10.1038/74155, 2-s2.0-0034028899. 10742093

[15] Dodhia S. , Celis K. , Aylward A. et al., ACSS2 Gene Variant Associated With Cleft Lip and Palate in Two Independent Hispanic Populations, The Laryngoscope. (2017) 127, no. 10, E336–E339, 10.1002/lary.26637, 2-s2.0-85029643449. 28543373 PMC5607083

[16] Mi N. , Hao Y. , Jiao X. et al., Association Study of Single Nucleotide Polymorphisms of MAFB With Non-Syndromic Cleft Lip With or Without Cleft Palate in a Population in Heilongjiang Province, Northern China, British Journal of Oral and Maxillofacial Surgery. (2014) 52, no. 8, 746–750, 10.1016/j.bjoms.2014.06.003, 2-s2.0-84920385813. 24972815

[17] Wang Q. , Sun S. , Song Q. , Hu H. , An J. , and Liu J. , The Risk of Nonsyndromic Cleft Lip with or Without Cleft Palate and Vax1 rs7078160 Polymorphisms in Southern Han Chinese, Brazilian Journal of Otorhinolaryngology. (2021) 87, no. 6, 718–722, 10.1016/j.bjorl.2020.08.007. 33132092 PMC9422622

[18] Marazita M. L. , The Evolution of Human Genetic Studies of Cleft Lip and Cleft Palate, Annual Review of Genomics and Human Genetics. (2012) 13, no. 1, 263–283, 10.1146/annurev-genom-090711-163729, 2-s2.0-84872450566. PMC3760163 22703175

[19] Cordell H. J. and Clayton D. G. , Genetic Association Studies, Lancet. (2005) 366, no. 9491, 1121–1131, 10.1016/s0140-6736(05)67424-7, 2-s2.0-25144501575. 16182901

[20] Biggs L. C. , Naridze R. L. , DeMali K. A. et al., Interferon Regulatory Factor 6 Regulates Keratinocyte Migration, Journal of Cell Science. (2014) 127, 2840–2848, 10.1242/jcs.139246, 2-s2.0-84903793174. 24777480 PMC4075356

[21] Carroll S. H. , Schafer S. , Dalessandro E. , Ho T. V. , Chai Y. , and Liao E. C. , Neural Crest and Periderm-Specific Requirements of Irf6 During Neural Tube and Craniofacial Development, Developmental Biology. (2025) 522, 106–115, 10.1016/j.ydbio.2025.03.006. 40113028 PMC12065081

[22] Thompson J. , Mendoza F. , Tan E. et al., A Cleft Lip and Palate Gene, Irf6, is Involved in Osteoblast Differentiation of Craniofacial Bone, Developmental Dynamics. (2019) 248, no. 3, 221–232, 10.1002/dvdy.13, 2-s2.0-85061276365. 30684382 PMC6414085

[23] Kondo S. , Schutte B. C. , Richardson R. J. et al., Mutations in IRF6 Cause Van Der Woude and Popliteal Pterygium Syndromes, Nature Genetics. (2002) 32, no. 2, 285–289, 10.1038/ng985, 2-s2.0-18644374446. 12219090 PMC3169431

[24] Zucchero T. M. , Cooper M. E. , Maher B. S. et al., Interferon Regulatory Factor 6 (IRF6) Gene Variants and the Risk of Isolated Cleft Lip or Palate, New England Journal of Medicine. (2004) 351, no. 8, 769–780, 10.1056/NEJMoa032909, 2-s2.0-4143115809. 15317890

[25] Alappat R. R. , Sachith S. K. , Varghese P. R. , Narayanan P. V. , and George A. , Parental Transmission Effects of the IRF6 Polymorphisms Among Non-Syndromic Cleft Lip With or Without Cleft Palate in Kerala Case Parent Trios, Archives of Oral Biology. (2025) 170, 10.1016/j.archoralbio.2024.106134. 39581118

[26] Zhou Q. , Li M. , Zhu W. et al., Association Between Interferon Regulatory Factor 6 Gene Polymorphisms and Nonsyndromic Cleft Lip With or Without Cleft Palate in a Chinese Population, The Cleft Palate-Craniofacial Journal. (2013) 50, no. 5, 570–576, 10.1597/12-234, 2-s2.0-84883650375. 23509905

[27] Pegelow M. , Koillinen H. , Magnusson M. et al., Association and Mutation Analyses of the IRF6 Gene in Families With Nonsyndromic and Syndromic Cleft Lip and/or Cleft Palate, The Cleft Palate-Craniofacial Journal. (2014) 51, no. 1, 49–55, 10.1597/11-220, 2-s2.0-84892391100. 23394314

[28] Avasthi K. K. , Agarwal A. , and Agarwal S. , Association of MTHFR, BMP4, TGFA and IRF6 Polymorphisms With Non-Syndromic Cleft Lip and Palate in North Indian Patients, Avicenna Journal of Medical Biotechnology. (2022) 14, 175–180, 10.18502/ajmb.v14i2.8879. 35633991 PMC9077655

[29] Gowans L. J. , Busch T. D. , Mossey P. A. et al., The Prevalence, Penetrance, and Expressivity of Etiologic IRF6 Variants in Orofacial Clefts Patients From Sub-Saharan Africa, Molecular Genetics & Genomic Medicine. (2017) 5, no. 2, 164–171, 10.1002/mgg3.273, 2-s2.0-85041394626. 28361103 PMC5370218

[30] Phan H. D. B. , Phuong L. H. , Dang T. N. , Tram D. B. , and Vu H. A. , Association of Single Nucleotide Polymorphisms in the IRF6 Gene with Nonsyndromic Cleft Lip With or Without Cleft Palate in Kinh Vietnamese Patients, Molecular Biology Reports. (2023) 50, no. 2, 1469–1476, 10.1007/s11033-022-08164-9. 36484949

[31] Zhang Z. , Song Y. , Zhao X. , Zhang X. , Fermin C. , and Chen Y. , Rescue of Cleft Palate in Msx1-Deficient Mice by Transgenic Bmp4 Reveals a Network of BMP and Shh Signaling in the Regulation of Mammalian Palatogenesis, Development. (2002) 129, no. 17, 4135–4146, 10.1242/dev.129.17.4135. 12163415

[32] Lidral A. C. , Romitti P. A. , Basart A. M. et al., Association of MSX1 and TGFB3 With Nonsyndromic Clefting in Humans, The American Journal of Human Genetics. (1998) 63, no. 2, 557–568, 10.1086/301956, 2-s2.0-0032231873. 9683588 PMC1377298

[33] Blanco R. , Jara L. , and Villaseca M. C. , Association of the Genetic Variation of (MSX1-7) and Non Syndromic Cleft Lip Palate in Chilean Subjects, Revista Medica de Chile. (1998) 126, no. 6, 637–645. 9778871

[34] Blanco R. , Jara L. , Villaseca C. , Palomino H. , and Carreno H. , Genetic Variation of MSX1 has a Sexual Dimorphism in Non Syndromic Cleft Palate in the Chilean Population, Revista Medica de Chile. (1998) 126, no. 7, 781–787. 9830770

[35] Jezewski P. A. , Vieira A. R. , Nishimura C. et al., Complete Sequencing Shows a Role for MSX1 in Non-Syndromic Cleft Lip and Palate, Journal of Medical Genetics. (2003) 40, no. 6, 399–407, 10.1136/jmg.40.6.399. 12807959 PMC1735501

[36] Butali A. , Mossey P. A. , Adeyemo W. L. et al., Genetic Studies in the Nigerian Population Implicate an MSX1 Mutation in Complex Oral Facial Clefting Disorders, The Cleft Palate-Craniofacial Journal. (2011) 48, no. 6, 646–653, 10.1597/10-133, 2-s2.0-80655144881. 21740177 PMC3206991

[37] Rashid R. , Rajion Z. A. , Zilfalil B. A. , and Jaafar S. , Association of rs8670 Polymorphism in the MSX1 Gene With Non-Syndromic Cleft Lip With or Without Cleft Palate in Malay Population, Cureus. (2024) 16, 10.7759/cureus.68958. PMC11461356 39385896

[38] Suzuki Y. , Jezewski P. A. , Machida J. et al., In a Vietnamese Population, MSX1 Variants Contribute to Cleft Lip and Palate, Genetics in Medicine. (2004) 6, no. 3, 117–125, 10.1097/01.gim.0000127275.52925.05, 2-s2.0-2642522952. 15354328

[39] Kamalakannan D. , Kailasam V. , Padmanaban S. , Paul S. F. D. , and Ramanathan G. , Association of MSX1 Gene Polymorphisms and Maxillary Lateral Incisor Agenesis in Non-Syndromic Cleft Lip and/or Palate Individuals, Journal of Oral Biology and Craniofacial Research. (2025) 15, no. 2, 440–443, 10.1016/j.jobcr.2025.02.003. 40092365 PMC11910350

[40] Jia S. , Zhou J. , Fanelli C. et al., Small-Molecule Wnt Agonists Correct Cleft Palates in Pax9 Mutant Mice in Utero, Development. (2017) 144, no. 20, 3819–3828, 10.1242/dev.157750, 2-s2.0-85032014634. 28893947 PMC5675451

[41] Yang C. W. , Shi J. Y. , Yin B. , Shi B. , and Jia Z. L. , Mutation at Paired Box Gene 9 is Associated With Non-Syndromic Cleft Lip Only From Western Han Chinese Population, Archives of Oral Biology. (2020) 117, 10.1016/j.archoralbio.2020.104829. 32679372

[42] Song T. , Wu D. , Wang Y. , Li H. , Yin N. , and Zhao Z. , SNPs and Interaction Analyses of IRF6, MSX1 and PAX9 Genes in Patients With Non-Syndromic Cleft Lip With or Without Palate, Molecular Medicine Reports. (2013) 8, no. 4, 1228–1234, 10.3892/mmr.2013.1617, 2-s2.0-84883344256. 23921572

[43] Romano R. A. , Solomon L. W. , and Sinha S. , Tp63 in Oral Development, Neoplasia, and Autoimmunity, Journal of Dental Research. (2012) 91, no. 2, 125–132, 10.1177/0022034511411302, 2-s2.0-84856400298. 21646640

[44] Yin B. , Shi J. Y. , Lin Y. S. , Shi B. , and Jia Z. L. , Snps at TP63 Gene was Specifically Associated With Right-Side Cleft Lip in Han Chinese Population, Oral Diseases. (2021) 27, no. 3, 559–566, 10.1111/odi.13566. 32687624

[45] Xu T. , Du M. , Bu X. et al., Identification of a Novel TP63 Mutation Causing Nonsyndromic Cleft Lip With or Without Cleft Palate, BMC Medical Genomics. (2021) 14, no. 1, 10.1186/s12920-021-00903-4. PMC7903685 33622322

[46] Abdulla R. , Kudkuli J. , Kapoor S. , Prabhu V. , Shetty P. , and Aziz N. Z. , Single-Nucleotide Polymorphisms of Methylenetetrahydrofolate Reductase Gene in a South Indian Cohort With Nonsyndromic Cleft Lip With or Without Palate, Journal of Oral and Maxillofacial Pathology. (2020) 24, no. 3, 453–458, 10.4103/jomfp.JOMFP_329_19. 33967480 PMC8083445

[47] Ma J. , Yin B. , Shi J. Y. et al., Exon Sequencing Reveals that Missense Mutation of PBX1 Gene may Increase the Risk of Non-Syndromic Cleft Lip/Palate, International Journal of Clinical and Experimental Pathology. (2019) 12, no. 7, 2691–2698. 31934099 PMC6949560

[48] Butali A. , Mossey P. , Adeyemo W. et al., Rare Functional Variants in Genome-Wide Association Identified Candidate Genes for Nonsyndromic Clefts in the African Population, American Journal of Medical Genetics, Part A. (2014) 164, no. 10, 2567–2571, 10.1002/ajmg.a.36691, 2-s2.0-84908228309. PMC4169286 25081408

[49] Chandrasekharan D. and Ramanathan A. , Identification of a Novel Heterozygous Truncation Mutation in Exon 1 of ARHGAP29 in an Indian Subject With Nonsyndromic Cleft Lip With Cleft Palate, European Journal of Dermatology. (2014) 8, no. 04, 528–532, 10.4103/1305-7456.143637, 2-s2.0-84916219219. PMC4253111 25512736

[50] Cui R. , Chen D. , Li N. et al., PARD3 Gene Variation as Candidate Cause of Nonsyndromic Cleft Palate Only, Journal of Cellular and Molecular Medicine. (2022) 26, no. 15, 4292–4304, 10.1111/jcmm.17452. 35789100 PMC9344820

[51] Gowans L. J. J. , Oseni G. , Mossey P. A. et al., Novel GREM1 Variations in Sub-Saharan African Patients With Cleft Lip and/or Cleft Palate, The Cleft Palate-Craniofacial Journal. (2018) 55, no. 5, 736–742, 10.1177/1055665618754948, 2-s2.0-85048578883. 29489415 PMC6081638

[52] Hao J. , Gao R. , Wu W. et al., Association Between BMP4 Gene Polymorphisms and Cleft Lip With or Without Cleft Palate in a Population From South China, Archives of Oral Biology. (2018) 93, 95–99, 10.1016/j.archoralbio.2018.05.015, 2-s2.0-85047763468. 29860186

[53] Tongkobpetch S. , Suphapeetiporn K. , Siriwan P. , and Shotelersuk V. , Study of the Poliovirus Receptor Related-1 Gene in Thai Patients With Non-Syndromic Cleft Lip With or Without Cleft Palate, International Journal of Oral and Maxillofacial Surgery. (2008) 37, no. 6, 550–553, 10.1016/j.ijom.2008.01.024, 2-s2.0-44249096793. 18356023

[54] Vogelaar I. P. , Figueiredo J. , van Rooij I. A. et al., Identification of Germline Mutations in the Cancer Predisposing Gene CDH1 in Patients With Orofacial Clefts, Human Molecular Genetics. (2013) 22, no. 5, 919–926, 10.1093/hmg/dds497, 2-s2.0-84873422090. 23197654

[55] Beaty T. H. , Murray J. C. , Marazita M. L. et al., A Genome-wide Association Study of Cleft Lip With and Without Cleft Palate Identifies Risk Variants Near MAFB and ABCA4, Nature Genetics. (2010) 42, no. 6, 525–529, 10.1038/ng.580, 2-s2.0-77952886672. 20436469 PMC2941216

[56] Lander E. S. , Linton L. M. , Birren B. et al., Initial Sequencing and Analysis of the Human Genome, Nature. (2001) 409, no. 6822, 860–921, 10.1038/35057062, 2-s2.0-2042437650. 11237011

[57] Metzker M. L. , Sequencing Technologies-The Next Generation, Nature Reviews Genetics. (2010) 11, no. 1, 31–46, 10.1038/nrg2626, 2-s2.0-72849144434. 19997069

[58] Heather J. M. and Chain B. , The Sequence of Sequencers: The History of Sequencing DNA, Genomics. (2016) 107, 1–8, 10.1016/j.ygeno.2015.11.003, 2-s2.0-84957436423. 26554401 PMC4727787

[59] Beck T. F. , Mullikin J. C. , and Biesecker L. G. , Systematic Evaluation of Sanger Validation of Next-Generation Sequencing Variants, Clinical Chemistry. (2016) 62, no. 4, 647–654, 10.1373/clinchem.2015.249623, 2-s2.0-84962374976. 26847218 PMC4878677

[60] Huang Y. F. , Chen S. C. , Chiang Y. S. , Chen T. H. , and Chiu K. P. , Palindromic Sequence Impedes Sequencing-by-Ligation Mechanism, BMC Systems Biology. (2012) 6, no. Suppl 2, 10.1186/1752-0509-6-S2-S10, 2-s2.0-84878657026. PMC3521181 23281822

[61] Hu T. , Chitnis N. , Monos D. , and Dinh A. , Next-Generation Sequencing Technologies: An Overview, Human Immunology. (2021) 82, no. 11, 801–811, 10.1016/j.humimm.2021.02.012. 33745759

[62] McCombie W. R. , McPherson J. D. , and Mardis E. R. , Next-Generation Sequencing Technologies, Cold Spring Harbor Perspectives in Medicine. (2019) 9, no. 11, 10.1101/cshperspect.a036798. PMC6824406 30478097

[63] Rabbani B. , Mahdieh N. , Hosomichi K. , Nakaoka H. , and Inoue I. , Next-Generation Sequencing: Impact of Exome Sequencing in Characterizing Mendelian Disorders, Journal of Human Genetics. (2012) 57, no. 10, 621–632, 10.1038/jhg.2012.91, 2-s2.0-84867946004. 22832387

[64] Beaty T. H. , Marazita M. L. , and Leslie E. J. , Genetic Factors Influencing Risk to Orofacial Clefts: Today’s Challenges and Tomorrow’s Opportunities, F1000Res. (2016) 5, 10.12688/f1000research.9503.1, 2-s2.0-85007607673. PMC5133690 27990279

[65] Albert T. J. , Molla M. N. , Muzny D. M. et al., Direct Selection of Human Genomic Loci by Microarray Hybridization, Nature Methods. (2007) 4, no. 11, 903–905, 10.1038/nmeth1111, 2-s2.0-35748951614. 17934467

[66] Gnirke A. , Melnikov A. , Maguire J. et al., Solution Hybrid Selection With Ultra-Long Oligonucleotides for Massively Parallel Targeted Sequencing, Nature Biotechnology. (2009) 27, no. 2, 182–189, 10.1038/nbt.1523, 2-s2.0-59849113821. PMC2663421 19182786

[67] Hodges E. , Rooks M. , Xuan Z. et al., Hybrid Selection of Discrete Genomic Intervals on Custom-Designed Microarrays for Massively Parallel Sequencing, Nature Protocols. (2009) 4, no. 6, 960–974, 10.1038/nprot.2009.68, 2-s2.0-66749159688. 19478811 PMC2990409

[68] Ng S. B. , Turner E. H. , Robertson P. D. et al., Targeted Capture and Massively Parallel Sequencing of 12 Human Exomes, Nature. (2009) 461, no. 7261, 272–276, 10.1038/nature08250, 2-s2.0-70249111091. 19684571 PMC2844771

[69] Voelkerding K. V. , Dames S. A. , and Durtschi J. D. , Next-Generation Sequencing: From Basic Research to Diagnostics, Clinical Chemistry. (2009) 55, no. 4, 641–658, 10.1373/clinchem.2008.112789, 2-s2.0-64149123778. 19246620

[70] Bartusel M. , Kim S. X. , Rehimi R. et al., A Non-Syndromic Orofacial Cleft Risk Locus Links tRNA Splicing Defects to Neural Crest Cell Pathologies, The American Journal of Human Genetics. (2025) 112, no. 5, 1097–1116, 10.1016/j.ajhg.2025.03.017. 40250422 PMC12120183

[71] Li M. J. , Shi J. Y. , Zhu Q. S. , Shi B. , and Jia Z. L. , Targeted Re-Sequencing of the 2p21 Locus Identifies Non-Syndromic Cleft Lip Only Novel Susceptibility Gene ZFP36L2, Frontiers in Genetics. (2022) 13, 10.3389/fgene.2022.802229. PMC8886408 35242166

[72] Li M. J. , Shi J. Y. , Zhang B. H. , Chen Q. M. , Shi B. , and Jia Z. L. , Targeted Re-Sequencing on 1p22 Among Non-Syndromic Orofacial Clefts From Han Chinese Population, Frontiers in Genetics. (2022) 13, 10.3389/fgene.2022.947126. PMC9428125 36061182

[73] Jia S. , Zhang S. , You Y. et al., Association Analysis Between Forkhead Box E1 Gene and Non-Syndromic Cleft Lip With or Without Cleft Palate in Han Chinese Population, Hua xi kou qiang yi xue za zhi. (2025) 43, no. 1, 28–36, 10.7518/hxkq.2024.2024110. 39840623 PMC11917515

[74] Lin Y. , Shi J. , Shi B. , and Jia Z. , Targeted Sequencing Reveals KLF4 Gene Associated With NSCL/P in Western Han Chinese, International Journal of Clinical and Experimental Pathology. (2019) 12, no. 10, 3894–3900. 31933779 PMC6949737

[75] Yang C. W. , You Y. , Sun J. L. , Shi B. , and Jia Z. L. , Integrated Analysis of the Association Between Variants at PAX7 and NSCL/P in the Han Population, The Cleft Palate-Craniofacial Journal. (2024) 61, no. 8, 1275–1282, 10.1177/10556656231163398. 36919448

[76] Tao H. X. , Yang Y. X. , Shi B. , and Jia Z. L. , Identification of Putative Regulatory Single-Nucleotide Variants in NTN1 Gene Associated with NSCL/P, Journal of Human Genetics. (2023) 68, no. 7, 491–497, 10.1038/s10038-023-01137-1. 36879001

[77] You Y. , Shi J. , Shi B. , and Jia Z. , Target Sequencing Reveals the Association Between Variants in VAX1 and NSCL/P in Chinese Population, Oral Diseases. (2023) 29, no. 5, 2130–2138, 10.1111/odi.14210. 35419918

[78] Zhao H. , He Q. , Wu X. et al., Identification of Rare Loss-of-function Variants in FAM3B Associated With Non-Syndromic Orofacial Clefts, Genomics. (2023) 115, no. 3, 10.1016/j.ygeno.2023.110630. 37105387

[79] Yang M. , Wang Y. , Yin B. , Zheng Q. , Shi B. , and Jia Z. , Association of Soluble Epoxide Hydrolase 2 Gene With the Risk of Non-Syndromic Cleft Lip With or Without Cleft Palate in Western Han Chinese Population, Hua Xi Kou Qiang Yi Xue Za Zhi. (2022) 40, no. 3, 279–284, 10.7518/hxkq.2022.03.005. 38597007 PMC9207791

[80] Dąbrowska J. , Biedziak B. , Bogdanowicz A. , and Mostowska A. , Identification of Novel Risk Variants of Non-Syndromic Cleft Palate by Targeted Gene Panel Sequencing, Journal of Clinical Medicine. (2023) 12, no. 5, 10.3390/jcm12052051. PMC10004578 36902838

[81] Choi M. , Scholl U. I. , Ji W. et al., Genetic Diagnosis by Whole Exome Capture and Massively Parallel DNA Sequencing, Proceedings of the National Academy of Sciences of the United States of America. (2009) 106, no. 45, 19096–19101, 10.1073/pnas.0910672106, 2-s2.0-73149123343. 19861545 PMC2768590

[82] Reddy H. M. , Cho K. A. , Lek M. et al., The Sensitivity of Exome Sequencing in Identifying Pathogenic Mutations for LGMD in the United States, Journal of Human Genetics. (2017) 62, no. 2, 243–252, 10.1038/jhg.2016.116, 2-s2.0-85010886965. 27708273 PMC5266644

[83] Ng S. B. , Buckingham K. J. , Lee C. et al., Exome Sequencing Identifies the Cause of a Mendelian Disorder, Nature Genetics. (2010) 42, no. 1, 30–35, 10.1038/ng.499, 2-s2.0-73349110071. 19915526 PMC2847889

[84] Clark M. J. , Chen R. , Lam H. Y. et al., Performance Comparison of Exome DNA Sequencing Technologies, Nature Biotechnology. (2011) 29, no. 10, 908–914, 10.1038/nbt.1975, 2-s2.0-80054757012. PMC4127531 21947028

[85] Bamshad M. J. , Ng S. B. , Bigham A. W. et al., Exome Sequencing as a Tool for Mendelian Disease Gene Discovery, Nature Reviews Genetics. (2011) 12, no. 11, 745–755, 10.1038/nrg3031, 2-s2.0-80054746492. 21946919

[86] Gilissen C. , Hehir-Kwa J. Y. , Thung D. T. et al., Genome Sequencing Identifies Major Causes of Severe Intellectual Disability, Nature. (2014) 511, no. 7509, 344–347, 10.1038/nature13394, 2-s2.0-84904465224. 24896178

[87] Yang Y. , Muzny D. M. , Reid J. G. et al., Clinical Whole-Exome Sequencing for the Diagnosis of Mendelian Disorders, New England Journal of Medicine. (2013) 369, no. 16, 1502–1511, 10.1056/NEJMoa1306555, 2-s2.0-84885785987. 24088041 PMC4211433

[88] Landoure G. , Sullivan J. M. , Johnson J. O. et al., Exome Sequencing Identifies a Novel TRPV4 Mutation in a CMT2C Family, Neurology. (2012) 79, no. 2, 192–194, 10.1212/WNL.0b013e31825f04b2, 2-s2.0-84866250052. 22675077 PMC3390542

[89] Alkharafi L. , Al-Bustan S. , Alkanderi S. et al., Identification of Novel and Rare Gene Variants in Cleft Lip/Palate Patients From Kuwaiti Consanguineous Families by Exome Sequencing, American Journal of Medical Genetics, Part A. (2025) 200, no. 2, e64268–e64370, 10.1002/ajmg.a.64268. 41041957

[90] Aladenika E. , Maskoen A. , Awotoye W. et al., Whole Exome Sequencing Identifies Damaging Variants in Indonesians with Clefts, The Cleft Palate-Craniofacial Journal. (2025) 62, no. 3, 460–465, 10.1177/10556656231210085. 37876262

[91] Zheng X. , Liang X. , Wu X. et al., Rare Variants in NRSN2 Cause Non-Syndromic Orofacial Cleft Through Dysregulation of TGF-Beta Signaling, Genes & Diseases. (2026) 13, no. 3, 10.1016/j.gendis.2025.101865. PMC12854861 41624910

[92] Genin E. , Missing Heritability of Complex Diseases: Case Solved?, Human Genetics. (2020) 139, no. 1, 103–113, 10.1007/s00439-019-02034-4, 2-s2.0-85067058182. 31165258

[93] Copelli M. M. , Atique-Tacla M. , Pairet E. et al., Whole Exome Sequencing in 18 Brazilian Families With Vertical Transmission of Non-Syndromic Oral Clefts, Journal of Cranio-Maxillo-Facial Surgery. (2025) 53, no. 4, 370–376, 10.1016/j.jcms.2024.12.016. 39855980

[94] Liang X. , He Q. , Jiao Y. et al., Identification of Rare Variants in PTCH2 Associated With Non-Syndromic Orofacial Clefts, Gene. (2024) 907, 10.1016/j.gene.2024.148280. 38360123

[95] Zhao H. , Zhong W. , Huang W. et al., Whole-Exome Sequencing Identifies ECPAS as a Novel Potentially Pathogenic Gene in Multiple Hereditary Families With Nonsyndromic Orofacial Cleft, Protein & Cell. (2024) 15, no. 10, 783–789, 10.1093/procel/pwae021. 38695759 PMC11443446

[96] Chen X. , Wang S. Y. , Xue E. C. et al., Exploring the Association Between De Novo Mutations and Non-Syndromic Cleft Lip With or Without Palate Based on Whole Exome Sequencing of Case-Parent Trios, Beijing Da Xue Xue Bao Yi Xue Ban. (2022) 54, no. 03.001, 387–393, 10.19723/j.issn.1671-167X.2022. 35701113 PMC9197716

[97] He Q. , Yu M. , Jiao Y. et al., Exome Sequencing Reveals the Genetic Architecture of Non-Syndromic Orofacial Clefts and Identifies BOC as a Novel Causal Gene, Advanced Science (Weinheim). (2025) 12, no. 32, 10.1002/advs.202412073. PMC12407381 40464334

[98] Zuo Y. , Chang J. W. , Zhong N. N. et al., Exome Analyses Unravel the Genetic Architecture of Mendelian Dominant Nonsyndromic Orofacial Clefts, Genomics. (2025) 117, no. 3, 10.1016/j.ygeno.2025.111039. 40147726

[99] Iovino E. , Scapoli L. , Palmieri A. et al., Ultra-Rare Variants Identify Biological Pathways and Candidate Genes in the Pathobiology of Non-Syndromic Cleft Palate Only, Biomolecules. (2023) 13, no. 2, 10.3390/biom13020236. PMC9953608 36830605

[100] Ranji P. , Pairet E. , Helaers R. et al., Four Putative Pathogenic ARHGAP29 Variants in Patients With Non-Syndromic Orofacial Clefts (NsOFC), European Journal of Human Genetics. (2025) 33, no. 1, 38–43, 10.1038/s41431-024-01727-3. 39506048 PMC11711499

[101] Zhao H. , Zhang M. , Zhong W. et al., A Novel IRF6 Mutation Causing Non-Syndromic Cleft Lip With or Without Cleft Palate in a Pedigree, Mutagenesis. (2018) 33, no. 3, 195–202, 10.1093/mutage/gey012, 2-s2.0-85056278540. 30053123

[102] Zhang M. , Huang J. , Shi F. et al., Identification of Pathogenic Variant in a Chinese Pedigree Affected With Non-Syndromic Cleft Lip and Palate, Zhonghua Yi Xue Yi Chuan Xue Za Zhi. (2021) 38, no. 1, 52–55, 10.3760/cma.j.cn511374-20200308-00144. 33423258

[103] Yu L. L. , Zeng Q. , Yu B. F. , Wei J. , and Dai C. C. , A Novel Genetic Variation Identified in Patients With Orofacial Clefts, Journal of Craniofacial Surgery. (2025) 36, no. 4, e437–e441, 10.1097/SCS.0000000000011011. 39679673

[104] Wang Y. , Ma C. , Jiang C. , Zhang Y. , and Wu D. , A Novel IRF6 Variant Detected in a Family With Nonsyndromic Cleft Lip and Palate by Whole Exome Sequencing, Journal of Craniofacial Surgery. (2021) 32, no. 1, 265–269, 10.1097/SCS.0000000000007000. 33136784

[105] Ding F. , Hou F. , Shan S. , Zhao Y. , and Jin H. , Case Report and Functional Verification of a Novel Mutation in the Interferon Regulatory Transcription Factor 6 Gene in a Family With Orofacial Clefts, American Journal of Translational Research. (2024) 16, no. 7, 2898–2909, 10.62347/IAQV2788. 39114717 PMC11301462

[106] Machado R. A. , Martelli-Junior H. , Reis S. R. A. et al., Identification of Novel Variants in Cleft Palate-Associated Genes in Brazilian Patients With Non-Syndromic Cleft Palate Only, Frontiers in Cell and Developmental Biology. (2021) 9, 10.3389/fcell.2021.638522. PMC8297955 34307341

[107] Hoebel A. K. , Drichel D. , van de Vorst M. et al., Candidate Genes for Nonsyndromic Cleft Palate Detected by Exome Sequencing, Journal of Dental Research. (2017) 96, no. 11, 1314–1321, 10.1177/0022034517722761, 2-s2.0-85029848781. 28767323

[108] Huang W. , He Q. , Li M. et al., Two Rare Variants Reveal the Significance of Grainyhead-Like 3 Arginine 391 Underlying Non-Syndromic Cleft Palate Only, Oral Diseases. (2023) 29, no. 4, 1632–1643, 10.1111/odi.14164. 35189007

[109] Belkadi A. , Bolze A. , Itan Y. et al., Whole-Genome Sequencing is More Powerful than Whole-Exome Sequencing for Detecting Exome Variants, Proceedings of the National Academy of Sciences of the United States of America. (2015) 112, no. 17, 5473–5478, 10.1073/pnas.1418631112, 2-s2.0-84928528797. 25827230 PMC4418901

[110] Maurano M. T. , Humbert R. , Rynes E. et al., Systematic Localization of Common Disease-Associated Variation in Regulatory DNA, Science. (2012) 337, no. 6099, 1190–1195, 10.1126/science.1222794, 2-s2.0-84865822182. 22955828 PMC3771521

[111] Akhtar-Zaidi B. , Cowper-Sal-lari R. , Corradin O. et al., Epigenomic Enhancer Profiling Defines a Signature of Colon Cancer, Science. (2012) 336, no. 6082, 736–739, 10.1126/science.1217277, 2-s2.0-84860841594. 22499810 PMC3711120

[112] Ward L. D. and Kellis M. , Interpreting Noncoding Genetic Variation in Complex Traits and Human Disease, Nature Biotechnology. (2012) 30, no. 11, 1095–1106, 10.1038/nbt.2422, 2-s2.0-84869436774. PMC3703467 23138309

[113] Yu Y. , Alvarado R. , Petty L. E. et al., Polygenic Risk Impacts PDGFRA Mutation Penetrance in Non-Syndromic Cleft Lip and Palate, Human Molecular Genetics. (2022) 31, no. 14, 2348–2357, 10.1093/hmg/ddac037. 35147171 PMC9307317

[114] Zhong X. , Han X. , Xie Q. et al., Identification of RESP18 Gene Mutations Linked to Hereditary Non-Syndromic Cleft Lip and Palate in a Southern Chinese Family, Medical Science Monitor. (2024) 30, 10.12659/MSM.944294. PMC11302214 38970244

[115] Awotoye W. , Mossey P. A. , Hetmanski J. B. et al., Whole-Genome Sequencing Reveals de-novo Mutations Associated With Nonsyndromic Cleft Lip/Palate, Scientific Reports. (2022) 12, no. 1, 10.1038/s41598-022-15885-1. PMC9273634 35817949

[116] Bishop M. R. , Diaz Perez K. K. , Sun M. et al., Genome-Wide Enrichment of De Novo Coding Mutations in Orofacial Cleft Trios, The American Journal of Human Genetics. (2020) 107, no. 1, 124–136, 10.1016/j.ajhg.2020.05.018. 32574564 PMC7332647

[117] Mukhopadhyay N. , Bishop M. , Mortillo M. et al., Whole Genome Sequencing of Orofacial Cleft Trios From the Gabriella Miller Kids First Pediatric Research Consortium Identifies a New Locus on Chromosome 21, Human Genetics. (2020) 139, no. 2, 215–226, 10.1007/s00439-019-02099-1. 31848685 PMC6981325

[118] Wilderman A. , VanOudenhove J. , Kron J. , Noonan J. P. , and Cotney J. , High-Resolution Epigenomic Atlas of Human Embryonic Craniofacial Development, Cell Reports. (2018) 23, no. 5, 1581–1597, 10.1016/j.celrep.2018.03.129, 2-s2.0-85045934354. 29719267 PMC5965702

[119] Lace B. , Pajusalu S. , Livcane D. et al., Monogenic Versus Multifactorial Inheritance in the Development of Isolated Cleft Palate: A Whole Genome Sequencing Study, Frontiers in Genetics. (2022) 13, 10.3389/fgene.2022.828534. PMC8907258 35281813

[120] Crane-Smith Z. , De Castro S. C. P. , Nikolopoulou E. et al., A Non-Coding Insertional Mutation of Grhl2 Causes Gene Over-Expression and Multiple Structural Anomalies Including Cleft Palate, Spina Bifida and Encephalocele, Human Molecular Genetics. (2023) 32, no. 17, 2681–2692, 10.1093/hmg/ddad094. 37364051 PMC10460492

[121] Abdellaoui A. , Yengo L. , Verweij K. J. H. , and Visscher P. M. , 15 Years of GWAS Discovery: Realizing the Promise, The American Journal of Human Genetics. (2023) 110, no. 2, 179–194, 10.1016/j.ajhg.2022.12.011. 36634672 PMC9943775

[122] Siu H. , Zhu Y. , Jin L. , and Xiong M. , Implication of Next-Generation Sequencing on Association Studies, BMC Genomics. (2011) 12, no. 1, 10.1186/1471-2164-12-322, 2-s2.0-79958800854. PMC3148210 21682891

[123] Gordeeva V. , Sharova E. , and Arapidi G. , Progress in Methods for Copy Number Variation Profiling, International Journal of Molecular Sciences. (2022) 23, no. 4, 10.3390/ijms23042143. PMC8879278 35216262

[124] Birnbaum R. , Slovik M. , Zenvirt S. et al., High Concordance of Copy Number Variants Detected by Chromosomal Microarray and Exome Sequencing in Clinical Diagnostics, Clinical Genetics. (2026) 109, no. 3, 529–538, 10.1111/cge.70079. 41014177 PMC12881219

[125] Guo X. , Delio M. , Haque N. et al., Variant Discovery and Breakpoint Region Prediction for Studying the Human 22q11.2 Deletion Using BAC Clone and Whole Genome Sequencing Analysis, Human Molecular Genetics. (2016) 25, no. 17, 3754–3767, 10.1093/hmg/ddw221, 2-s2.0-85014371970. 27436579 PMC5216616

[126] van Dijk E. L. , Naquin D. , Gorrichon K. et al., Genomics in the Long-Read Sequencing Era, Trends in Genetics. (2023) 39, no. 9, 649–671, 10.1016/j.tig.2023.04.006. 37230864

[127] Rhoads A. and Au K. F. , PacBio Sequencing and Its Applications, Genomics, Proteomics & Bioinformatics. (2015) 13, no. 5, 278–289, 10.1016/j.gpb.2015.08.002, 2-s2.0-84947299022. PMC4678779 26542840

[128] Patel A. , Schwab R. , Liu Y. T. , and Bafna V. , Amplification and Thrifty Single-Molecule Sequencing of Recurrent Somatic Structural Variations, Genome Research. (2014) 24, no. 2, 318–328, 10.1101/gr.161497.113, 2-s2.0-84893641770. 24307551 PMC3912422

[129] Friedman N. and Rando O. J. , Epigenomics and the Structure of the Living Genome, Genome Research. (2015) 25, no. 10, 1482–1490, 10.1101/gr.190165.115, 2-s2.0-84942922295. 26430158 PMC4579333

[130] Howe L. J. , Richardson T. G. , Arathimos R. et al., Evidence for DNA Methylation Mediating Genetic Liability to Non-Syndromic Cleft Lip/Palate, Epigenomics. (2019) 11, no. 2, 133–145, 10.2217/epi-2018-0091, 2-s2.0-85061284293. 30638414 PMC6462847

[131] Charoenvicha C. , Sirimaharaj W. , Khwanngern K. , Chattipakorn N. , and Chattipakorn S. C. , Alterations in DNA Methylation in Orofacial Clefts, International Journal of Molecular Sciences. (2022) 23, no. 21, 10.3390/ijms232112727. PMC9654384 36361518

[132] Alvizi L. , Ke X. , Brito L. A. et al., Differential Methylation is Associated With Non-Syndromic Cleft Lip and Palate and Contributes to Penetrance Effects, Scientific Reports. (2017) 7, no. 1, 10.1038/s41598-017-02721-0, 2-s2.0-85019723691. PMC5446392 28550290

[133] Schwartzman O. and Tanay A. , Single-Cell Epigenomics: Techniques and Emerging Applications, Nature Reviews Genetics. (2015) 16, no. 12, 716–726, 10.1038/nrg3980, 2-s2.0-84947617259. 26460349

[134] Zhang B. , Zhang Y. , Wu S. , Ma D. , and Ma J. , DNA Methylation Profile of Lip Tissue From Congenital Nonsyndromic Cleft Lip and Palate Patients by Whole-Genome Bisulfite Sequencing, Birth Defects Res. (2023) 115, no. 2, 205–217, 10.1002/bdr2.2102. 36210532 PMC10092010

[135] Young J. I. , Slifer S. , Hecht J. T. , and Blanton S. H. , DNA Methylation Variation Is Identified in Monozygotic Twins Discordant for Non-Syndromic Cleft Lip and Palate, Frontiers in Cell and Developmental Biology. (2021) 9, 10.3389/fcell.2021.656865. PMC8149607 34055787

[136] Liu S. , Higashihori N. , Yahiro K. , and Moriyama K. , Retinoic Acid Inhibits Histone Methyltransferase Whsc1 During Palatogenesis, Biochemical and Biophysical Research Communications. (2015) 458, no. 3, 525–530, 10.1016/j.bbrc.2015.01.148, 2-s2.0-84924032180. 25677622

[137] Yuan X. , Qiu L. , Pu Y. et al., Histone Acetylation is Involved in TCDD-Induced Cleft Palate Formation in Fetal Mice, Molecular Medicine Reports. (2016) 14, no. 2, 1139–1145, 10.3892/mmr.2016.5348, 2-s2.0-84978397956. 27279340 PMC4940082

[138] Grandi F. C. , Modi H. , Kampman L. , and Corces M. R. , Chromatin Accessibility Profiling by ATAC-Seq, Nature Protocols. (2022) 17, no. 6, 1518–1552, 10.1038/s41596-022-00692-9. 35478247 PMC9189070

[139] Lee K. H. , Kim J. , and Kim J. H. , 3D Epigenomics and 3D Epigenopathies, BMB Reports. (2024) 57, no. 5, 216–231, 10.5483/BMBRep.2023-0249. 38627948 PMC11139681

[140] Lou S. , Yang J. , Zhu G. R. et al., Integrative Multi-Omics Analysis Identifies Genetic Variants Contributing to Non-Syndromic Cleft Lip With or Without Cleft Palate, Chinese Journal of Dental Research. (2024) 27, no. 1, 65–73, 10.3290/j.cjdr.b5136745. 38546521

[141] Xiao Y. , Jiao S. , He M. et al., Chromatin Conformation of Human Oral Epithelium Can Identify Orofacial Cleft Missing Functional Variants, International Journal of Oral Science. (2022) 14, no. 1, 10.1038/s41368-022-00194-0. PMC9411193 36008388

[142] Wang K. C. and Chang H. Y. , Epigenomics: Technologies and Applications, Circulation Research. (2018) 122, no. 9, 1191–1199, 10.1161/CIRCRESAHA.118.310998, 2-s2.0-85051509564. 29700067 PMC5929475

[143] Shull L. C. and Artinger K. B. , Epigenetic Regulation of Craniofacial Development and Disease, Birth Defects Res. (2024) 116, no. 1, 10.1002/bdr2.2271. PMC10872612 37964651

[144] Lei Y. , Tang R. , Xu J. et al., Applications of Single-Cell Sequencing in Cancer Research: Progress and Perspectives, Journal of Hematology & Oncology. (2021) 14, no. 1, 10.1186/s13045-021-01105-2. PMC8190846 34108022

[145] Hwang B. , Lee J. H. , and Bang D. , Single-Cell RNA Sequencing Technologies and Bioinformatics Pipelines, Experimental and Molecular Medicine. (2018) 50, no. 8, 1–14, 10.1038/s12276-018-0071-8, 2-s2.0-85060183020. PMC6082860 30089861

[146] Wu J. , Ding Y. , Wang J. et al., Single-Cell RNA Sequencing in Oral Science: Current Awareness and Perspectives, Cell Proliferation. (2022) 55, no. 10, 10.1111/cpr.13287. PMC9528768 35842899

[147] Kim D. S. , ATAC-Seq Data Processing, Methods in Molecular Biology. (2023) 2611, 305–323, 10.1007/978-1-0716-2899-7_17. 36807076

[148] Cai S. and Yin N. , Single-Cell Transcriptome and Chromatin Accessibility Mapping of Upper Lip and Primary Palate Fusion, Journal of Cellular and Molecular Medicine. (2024) 28, no. 19, 10.1111/jcmm.70128. PMC11467802 39392189

[149] Huang W. , Qian Z. , Zhang J. et al., A Single-Cell Atlas of Developing Mouse Palates Reveals Cellular and Molecular Transitions in Periderm Cell Fate, Genomics, Proteomics & Bioinformatics. (2025) 23, no. 1, 10.1093/gpbjnl/qzaf013. PMC12240470 40037804

[150] Ozekin Y. H. , O’Rourke R. , and Bates E. A. , Single Cell Sequencing of the Mouse Anterior Palate Reveals Mesenchymal Heterogeneity, Developmental Dynamics. (2023) 252, no. 6, 713–727, 10.1002/dvdy.573. 36734036 PMC10238667

[151] Sun B. , Reynolds K. , Saha S. K. , Zhang S. , McMahon M. , and Zhou C. J. , Ezh2-Dependent Methylation in Oral Epithelia Promotes Secondary Palatogenesis, Birth Defects Research. (2023) 115, no. 19, 1851–1865, 10.1002/bdr2.2216. 37435868 PMC10784412

[152] Itai T. , Yan F. , Liu A. et al., Investigating Gene Functions and Single-Cell Expression Profiles of De Novo Variants in Orofacial Clefts, HGG Advance. (2024) 5, no. 3, 10.1016/j.xhgg.2024.100313. PMC11318074 38807368

[153] Ozekin Y. H. , O’Rourke R. , and Bates E. A. , Kcnj2 Regulates Electrical Activity-Induced Gene Networks in Embryonic Mouse Palate Shelves, Developmental Biology. (2025) 527, 260–274, 10.1016/j.ydbio.2025.07.019. 40783019

[154] Wang B. , Xu M. , Zhao J. , Yin N. , Wang Y. , and Song T. , Single-Cell Transcriptomics Reveals Activation of Macrophages in All-trans Retinoic Acid (atRA)-induced Cleft Palate, Journal of Craniofacial Surgery. (2024) 35, no. 1, 177–184, 10.1097/SCS.0000000000009782. 38049149

[155] Siewert A. , Hoeland S. , Mangold E. , and Ludwig K. U. , Combining Genetic and Single-Cell Expression Data Reveals Cell Types and Novel Candidate Genes for Orofacial Clefting, Scientific Reports. (2024) 14, no. 1, 10.1038/s41598-024-77724-9. PMC11532359 39489835

[156] Gu Y. , Hu Y. , Zhang H. , Wang S. , Xu K. , and Su J. , Single-Cell RNA Sequencing in Osteoarthritis, Cell Proliferation. (2023) 56, no. 12, 10.1111/cpr.13517. PMC10693192 37317049

[157] Kulasinghe A. , Berrell N. , Donovan M. L. , and Nilges B. S. , Spatial-Omics Methods and Applications, Methods in Molecular Biology. (2025) 2880, 101–146, 10.1007/978-1-0716-4276-4_5. 39900756

[158] Wang J. , Ye F. , Chai H. et al., Advances and Applications in Single-Cell and Spatial Genomics, Science China Life Sciences. (2025) 68, no. 5, 1226–1282, 10.1007/s11427-024-2770-x. 39792333

[159] Wang B. , Zhang Z. , Zhao J. et al., Spatiotemporal Evolution of Developing Palate in Mice, Journal of Dental Research. (2024) 103, no. 5, 546–554, 10.1177/00220345241232317. 38619065 PMC11145300

[160] Pina J. O. , Raju R. , Roth D. M. et al., Multimodal Spatiotemporal Transcriptomic Resolution of Embryonic Palate Osteogenesis, Nature Communications. (2023) 14, no. 1, 10.1038/s41467-023-41349-9. PMC10502152 37709732

[161] Jain S. and Eadon M. T. , Spatial Transcriptomics in Health and Disease, Nature Reviews Nephrology. (2024) 20, no. 10, 659–671, 10.1038/s41581-024-00841-1. 38719971 PMC11392631

[162] Williams C. G. , Lee H. J. , Asatsuma T. , Vento-Tormo R. , and Haque A. , An Introduction to Spatial Transcriptomics for Biomedical Research, Genome Medicine. (2022) 14, no. 1, 10.1186/s13073-022-01075-1. PMC9238181 35761361

[163] Baysoy A. , Bai Z. , Satija R. , and Fan R. , The Technological Landscape and Applications of Single-Cell Multi-Omics, Nature Reviews Molecular Cell Biology. (2023) 24, no. 10, 695–713, 10.1038/s41580-023-00615-w. 37280296 PMC10242609

[164] Babu M. and Snyder M. , Multi-Omics Profiling for Health, Molecular and Cellular Proteomics. (2023) 22, no. 6, 10.1016/j.mcpro.2023.100561. PMC10220275 37119971

[165] Yan F. , Suzuki A. , Iwaya C. et al., Single-Cell Multiomics Decodes Regulatory Programs for Mouse Secondary Palate Development, Nature Communications. (2024) 15, no. 1, 10.1038/s41467-024-45199-x. PMC10821874 38280850

[166] Pina J. O. , Raju R. , Roth D. M. et al., Spatial Multi-Omics Reveals the Role of the Wnt Modulator, Dkk2, in Palatogenesis, Journal of Dental Research. (2024) 103, no. 13, 1412–1420, 10.1177/00220345241256600. 38910391 PMC11653329

[167] Hayes C. N. , Nakahara H. , Ono A. , Tsuge M. , and Oka S. , From Omics to Multi-Omics: A Review of Advantages and Tradeoffs, Genes. (2024) 15, no. 12, 10.3390/genes15121551. PMC11675490 39766818

[168] Cui H. , Wang C. , Maan H. et al., scGPT: Toward Building a Foundation Model for Single-Cell Multi-Omics Using Generative AI, Nature Methods. (2024) 21, no. 8, 1470–1480, 10.1038/s41592-024-02201-0. 38409223

[169] Thieme F. , Henschel L. , Hammond N. L. et al., Extending the Allelic Spectrum at Noncoding Risk Loci of Orofacial Clefting, Human Mutation. (2021) 42, no. 8, 1066–1078, 10.1002/humu.24219. 34004033

[170] Lewis C. M. and Vassos E. , Polygenic Risk Scores: From Research Tools to Clinical Instruments, Genome Medicine. (2020) 12, no. 1, 10.1186/s13073-020-00742-5. PMC7236300 32423490

[171] Mavaddat N. , Michailidou K. , Dennis J. et al., Polygenic Risk Scores for Prediction of Breast Cancer and Breast Cancer Subtypes, The American Journal of Human Genetics. (2019) 104, no. 1, 21–34, 10.1016/j.ajhg.2018.11.002, 2-s2.0-85059498503. 30554720 PMC6323553

[172] Zhu M. , Zhu X. , Han Y. et al., Polygenic Risk Scores for Pan-Cancer Risk Prediction in the Chinese Population: A Population-Based Cohort Study Based on the China Kadoorie Biobank, PLoS Medicine. (2025) 22, no. 2, 10.1371/journal.pmed.1004534. PMC11870365 40019942

[173] Patel A. P. , Wang M. , Ruan Y. et al., A Multi-Ancestry Polygenic Risk Score Improves Risk Prediction for Coronary Artery Disease, Nature Medicine. (2023) 29, no. 7, 1793–1803, 10.1038/s41591-023-02429-x. PMC10353935 37414900

[174] Kang G. , Baek S. H. , Kim Y. H. , Kim D. H. , and Park J. W. , Genetic Risk Assessment of Nonsyndromic Cleft Lip With or Without Cleft Palate by Linking Genetic Networks and Deep Learning Models, International Journal of Molecular Sciences. (2023) 24, no. 5, 10.3390/ijms24054557. PMC10003462 36901988

[175] Wang S. , Shi J. , Liu C. et al., Evidence of the Folate-Mediated One-Carbon Metabolism Pathway Genes in Controlling the Non-Syndromic Oral Clefts Risks, Oral Diseases. (2023) 29, no. 3, 1080–1088, 10.1111/odi.14068. 34739175

[176] Ma L. , Lou S. , Miao Z. et al., Identification of Novel Susceptibility Loci for Non-Syndromic Cleft Lip With or Without Cleft Palate, Journal of Cellular and Molecular Medicine. (2020) 24, no. 23, 13669–13678, 10.1111/jcmm.15878. 33108691 PMC7754035

[177] Zhang S. J. , Meng P. , Zhang J. et al., Machine Learning Models for Genetic Risk Assessment of Infants With Non-Syndromic Orofacial Cleft, Genomics, Proteomics & Bioinformatics. (2018) 16, no. 5, 354–364, 10.1016/j.gpb.2018.07.005, 2-s2.0-85059175848. PMC6364041 30578914

[178] Pan H. , Liu Z. , Ma J. et al., Genome-Wide Association Study Using Whole-Genome Sequencing Identifies Risk Loci for Parkinson’s disease in Chinese Population, NPJ Parkinson’s Disease. (2023) 9, no. 1, 10.1038/s41531-023-00456-6. PMC9911746 36759515

[179] Arni A. M. , Fraser D. P. , Sharp S. A. et al., Type 1 Diabetes Genetic Risk Score Variation Across Ancestries Using Whole Genome Sequencing and Array-Based Approaches, Scientific Reports. (2024) 14, 10.1038/s41598-024-82278-x. PMC11680773 39730838

